# Supplementary material for: Assembly of transmembrane pores from mirror-image peptides
Source: Nat Commun. 2022 Sep 14;13:5377. doi: 10.1038/s41467-022-33155-6 (PMC9474448; doi:10.1038/s41467-022-33155-6)
Supplement: Supplementary file 1 — Supplementary Information [file 41467_2022_33155_MOESM1_ESM.pdf]

Supplementary Information

Assembly of Transmembrane Pores from Mirror-Image Peptides

Smrithi Krishnan R[1,2†], Kalyanashis Jana[3†], Amina H Shaji[1†], Karthika S Nair[4,5], Anjali Devi Das[1], Devika Vikraman[1,2], Harsha Bajaj[4,5], Ulrich Kleinekathöfer[3], and Kozhinjampara R Mahendran[1]*

[†]These authors contributed equally

[1]Membrane Biology Laboratory, Transdisciplinary Research Program, Rajiv Gandhi Centre for Biotechnology, Thiruvananthapuram 695014, India.

[2]Manipal Academy of Higher Education, Manipal, Karnataka, India-576104

[3]Department of Physics and Earth Sciences, Jacobs University Bremen, 28759 Bremen, Germany

[4]Microbial Processes and Technology Division, CSIR- National Institute for Interdisciplinary Science and Technology (NIIST), Thiruvananthapuram 695019, India

[5]Academy of Scientific and Innovative Research (AcSIR), CSIR-Human Resource Development Centre, Ghaziabad 201002, India

*To whom correspondence should be addressed

*e-mail: mahendran@rgcb.res.in

Table of Contents

Supplementary Materials 4

Supplementary Text 4

a) Gel extraction and purification of DpPorA preoligomers 4

b) Ion selectivity measurements through DpPorA 5

c) Proteinase K enzyme activity on L and D pores 5

d) Transport across giant vesicles in the presence of proteinase K and data analysis 6

e) Molecular dynamics simulations of L and D pores 7

f) Applied-field MD simulations 8

g) Structural insights of LcWza and DcWza pores 8

h) Ramachandran Plot of the modeled pores 9

Supplementary Tables 10

a) Table 1: Nonaarginine binding kinetics with DpPorA 10

b) Table 2: Average computed conductance values (GMD) for L and D pores 11

Supplementary Figures 12

Fig. 1: HPLC traces and mass spectra for DpPorA peptide. 12

Fig. 2: Electrical recordings of gel extracted DpPorA (S) 13

Fig. 3: Electrical recordings of gel extracted DpPorA (L) 14

Fig. 4: Electrical properties of DpPorA at different salt conditions and comparison with LpPorA 15

Fig. 5: Interaction of DpPorA with cationic am$_8\gamma$CD 16

Fig. 6: Interaction of DpPorA with differently charged cyclic sugars 17

Fig. 7: Interaction of DpPorA with differently charged peptides 18

Fig. 8: Interaction of DpPorA with PEG polymers 19

Fig. 9: Functional stability of DpPorA and LpPorA to protease reaction 20

Fig. 10: Functional assembly of LpPorA in giant vesicles 21

Fig. 11: Biophysical and electrical properties of transient DcWza 22

Fig. 12: Electrical properties of DcWza and transport in giant vesicle system 23

Fig. 13: Comparison of the electrical properties of D and LcWza and functional stability of D and LcWza to Proteinase K 24

Fig. 14: Protein backbone-backbone RMSD values with respect to the

initial pPorA structures for 200 ns unbiased MD simulations 25

Fig. 15: Final pPorA conformations 26

Fig. 16: Cationic $am_8\gamma CD$ bound to L and DpPorA 27

Fig. 17: Protein backbone-backbone RMSD values with respect to the initial cWza structures for 200 ns unbiased MD simulations 29

Fig. 18: Final L and DcWza conformations 31

Fig. 19: Crystal structure of the α-helical outer membrane D4 domain of Wza compared to the designed cWza structure 32

Fig. 20: Radius along the channel axis and electrostatic potential of the DcWza and LcWza 33

Fig. 21: Ramachandran plots of the modeled pores 34

Supplementary References 35

Source data for SDS gels 36

Supplementary Materials:

The following materials were used for the study: 1,2-diphytanoyl-*sn*-glycero-3-phosphocholine (DPhPC, Avanti Polar Lipids), pentane (Sigma-Aldrich Merck), hexadecane (Sigma-Aldrich Merck), n-dodecyl β-D-maltoside (DDM, Sigma-Aldrich Merck), potassium chloride (Sigma-Aldrich Merck), 4-(2-hydroxyethyl)-1-piperazineethanesulfonic acid (HEPES, Sigma-Aldrich Merck), ethylenediaminetetraacetic acid disodium salt (EDTA, Sigma-Aldrich Merck), dithiothreitol (DTT, Sigma-Aldrich Merck), 2-propanol (Sigma-Aldrich Merck), methanol (Sigma-Aldrich Merck), octakis-(6-amino-6-deoxy)-γ-cyclodextrin octahydrochloride ($am_8\gamma CD$, AraChem Cyclodextrin-Shop), proteinase K (Sigma-Aldrich Merck), phenylmethylsulfonyl fluoride (PMSF, Sigma-Aldrich Merck), 2x Laemmli sample buffer (Bio-Rad), Any kD™ Mini-PROTEAN® TGX™ precast gel (Bio-Rad), Precision Plus Protein™ Dual Color Standards (Bio-Rad), ATTO-550 DOPE, Mowiol 28-99 (MW 145,000 Da fully hydrolyzed polyvinyl alcohol PVA) all other reagents (Sigma-Aldrich Merck). Alexa Fluor 350 hydrazide (Thermo-Fischer Invitrogen). monomethoxy poly(ethylene glycol)-*o*-pyridyl disulfide, MW 1000 (MePEG-OPSS, Nanocs). The peptides were purchased from Peptide Protein Research Ltd and GaloreTx Pharmaceuticals Private Limited, at >95% purity (HPLC) as lyophilized powders.

Supplementary Text:

a) Gel extraction and purification of DpPorA preoligomers

The DpPorA peptides used in this study were purchased from GaloreTx Pharmaceuticals Private Limited, Bangalore, India. The peptides were synthesized in a linear fashion using solid-phase peptide synthesis. All raw materials used for the synthesis of the D-peptide had chiral purity greater than 98%. Preparative high-performance liquid chromatography (HPLC) was conducted to purify the synthesized peptide. Reversed-phase HPLC was conducted using an Aeris™ 3.6 μm PEPTIDE XB-C18, 250 mm × 4.6 mm (flow 1 mL min^{-1}) column as stationary phase and a gradient of acetonitrile in water with 0.1 % TFA as mobile phase. A purity of greater than 97% was achieved for the target peptide. The HPLC trace and mass spectrum is provided in Figure S1.

SDS-polyacrylamide gel electrophoresis (SDS-PAGE) to identify and purify the DpPorA peptide preoligomers. The HPLC purified peptides were solubilized in 10 mM phosphate buffer

containing 0.1% DDM (pH 7.4). The peptide samples were mixed with 4x Laemmli sample buffer (Bio-Rad) and loaded on a Mini-PROTEAN® TGX™ precast any kDa protein gel (Bio-Rad) with Protein Standard Marker (Precision Plus Protein™ Dual Xtra prestained protein standards) (Bio-Rad) followed by SDS-PAGE. The electrophoretic mobility of peptides in SDS-PAGE was determined, and molecular mass was calculated based on denatured protein standards. Precisely, bands containing corresponding DpPorA preoligomers (~ 35 kDa) were cut from the gel, and each gel slice was solubilized with 10 mM phosphate buffer (K_2HPO_4, KH_2PO_4, pH 7.4, 500 µL) and crushed with a plastic pestle and cut into tiny pieces. After 30 min at room temperature, the gel fragments were removed with microfilterfuge tubes by centrifugation at 15,000 ×g. Finally, the filtrate containing the DpPorA preoligomers were used to examine the pore-forming activity in lipid bilayers using electrical recordings.

b) Ion selectivity measurements through DpPorA

The ion selectivity measurements were performed by establishing a KCl concentration gradient across the bilayer chamber (1 M KCl, cis and 0.15 M KCl, trans). The potential difference was applied through Ag/AgCl electrodes with agarose salt bridges. The pore formation in the membrane resulted in current at 0 mV. Subsequently, this current was manually set to zero by adjusting the applied voltage. The voltage required to achieve zero current was referred to as the 'reverse potential' (V_m), which can be used to calculate the permeability ratio of K^+ and Cl^- ions across the pore. The ion selectivity of the pores has been characterized by the Goldman-Hodgkin-Katz equation[1].

$$V_m = \frac{RT}{F}\ln\left(\frac{P_{K^+}[K^+]^{cis} + P_{Cl^-}[Cl^-]^{trans}}{P_{K^+}[K^+]^{trans} + P_{Cl^-}[Cl^-]^{cis}}\right) \qquad (1)$$

In this equation, *R* is the universal gas constant (8.314 $J.K^{-1}.mol^{-1}$), *T* is the temperature in Kelvin (K = °C + 273.15), *F* is Faraday's constant (96485 $C.mol^{-1}$), P_K is the membrane permeability for K^+, P_{Cl} is the relative membrane permeability for Cl^-, $[K^+]^{cis}$ is the concentration of K^+ in the cis side, $[K^+]^{trans}$ is the concentration of K^+ in the trans side, $[Cl^-]^{cis}$ is the concentration of Cl^- in the cis side and $[Cl^-]^{trans}$ is the concentration of Cl^- in the trans side. The reverse potential for DpPorA was calculated to be +32 mV.

c) Proteinase K enzyme activity on L and D pores

LpPorA, DpPorA, LcWza and DcWza peptides were prepared in 10 mM phosphate buffer with 0.1% DDM (pH 7.4). A final concentration of 0.5 mg/ml freshly prepared proteinase K solution

was added to the samples. The reaction mixtures were heated at 63° C for 30 min. The proteolysis was terminated by adding 2 mM PMSF (phenylmethyl sulfonyl fluoride)[2]. The reaction products were loaded on a Mini-PROTEAN® TGX™ precast gel (Bio-Rad) with Protein Standard Marker (Bio-Rad) followed by SDS-PAGE. The proteolysis products were analyzed along with the proteinase K untreated peptide samples as a control. We observed that while both the L peptides underwent complete proteolytic degradation, the D peptides remained unaffected. A higher molecular weight band of ~70 kDa corresponding to PMSF was observed upon the SDS-PAGE of proteinase K and PMSF treated samples. The reaction products were tested for pore formation in single-channel electrical recordings.

d) Transport across giant vesicles in the presence of proteinase K and data analysis

The peptide DpPorA was treated with proteinase K (0.5 mg/ml) at 63° C for 30 min, followed by the addition of 2 mM PMSF (phenylmethyl sulfonyl fluoride) to terminate the reaction. The mixture is kept at room temperature for 5 min. The treated peptide was then added to the GUV sample and incubated for 15 min, at the end of which Alexa-Fluor 350 dye was added to the vesicles and imaged to calculate vesicle permeabilization rates for statistical analysis. Control vesicles were subjected to the same concentration of detergent, Proteinase K, and PMSF and imaged in the presence of Alexa Fluor 350. The vesicle intensity (I_{in}) was quantified and normalized against the vesicle intensity outside(I_{out}) in each vesicle to determine the percentage of complete permeabilization by proteinase K treated DpPorA in vesicles.

Data analysis: The fluorescent intensity quantification is performed on uneven illumination subtracted images as described in ImageJ/FIJI. An average of ten 18 × 18 square pixels is created to determine the intensity outside the vesicles (I_{out}) and one 18 × 18-pixel box inside the vesicle to calculate the intensity inside the vesicle (I_{in}) at respective times. The vesicles are considered completely permeabilized when $I_{in} \approx I_{out}$. We set the threshold for completely permeabilized vesicles as $I_{in} / I_{out} > 0.99$. For the statistical analysis, the percentage of completely permeabilized vesicle population $I_{in} / I_{out} > 0.99$ is determined after 35 min of incubation with peptide/detergent and fluorescent dye. The time dependence curve of normalized intensity is calculated from individual vesicles mean values and corresponding standard errors. This percentage is compared to the vesicles in the presence and absence of respective peptides to understand the pore-forming properties of peptides. The mean and

standard deviation of completely permeabilized vesicles are calculated from respective independent batches.

Data acquisition - The images of giant unilamellar vesicles were acquired using Carl Zeiss Axio imager2 upright microscope with an epifluorescence module. The Carl Zeiss microscope employs a 2.83 MP Axiocam 503 monochrome with 1936 (H) × 1460 (V) sensor pixel count. A 40x (NA 0.75) objective was used to image the transport of molecules across DpPorA. The lipids and fluorescent dye Alexa-Fluor 350 were imaged in TRITC and DAPI filters.

e) Molecular dynamics simulations of L and D pores

The modeled pores were embedded into a 1,2-diphytanoyl-sn-glycero-3-phosphocholine (DPhPC) bilayer and solvated with TIP3P water molecules on both sides of the membrane, maintaining a water thickness of 25 Å on each side of the membrane using the CHARMM-GUI Membrane Builder[3]. After neutralizing the charge of the channels by adding K^+ or Cl^- ions, 1.0 M KCl was added to mimic the experimental conditions and to explore the ion conductance of the channels. Subsequently, the binding of a cationic gamma cyclodextrin ($am_8\gamma CD$) molecule to a pPorA pores was investigated. Force field parameters for the CD were available[4]. All systems composed of the protein, membrane, solvent, ions, and ligand were placed in a rectangular box of approximately 8.5 × 8.5 × 10 nm^3 containing roughly 85000 atoms. We have performed all the MD simulations with the CHARMM36-m forcefield using GROMACS-5.1.14[5,6].

The steepest descent method was used for the energy minimization of the model systems. A two-step constant volume (NVT) equilibration of 5 ns each with varying restrains on the simulated system at 300 K was employed using a Berendsen thermostat with a temperature coupling constant of 1 ps. After that, the systems were relaxed in a four-step constant pressure (NPT) equilibration of 75 ns by removing the restraints on the protein, the membrane, and the ligands in a stepwise manner using the semi-isotropic coupling method to a Berendsen barostat at 1 bar with a coupling constant of 2 ps. The Verlet cut-off scheme was applied for Coulomb and Lennard-Jones interactions with a cut-off of 10 Å to consider nonbonded interactions. In contrast, the Particle Mesh Ewald scheme was employed to evaluate long-range electrostatic interactions[7]. All bonds in the proteins were constrained using the Linear Constraint Solver (LINCS) algorithm[8]. The six-step equilibrated systems were subsequently simulated three times for 200 ns each in an NPT ensemble using a Parrinello-Rahman barostat along with a

semi-isotropic pressure coupling and the Nose-Hover thermostat[9]. One of the simulations for each was further extended to 500 ns. The root mean square deviations from the initial structure (RMSD) were calculated to examine the system stability and structural changes of the designed channels. The final structures resulting from the unbiased MD simulations after 200 ns were further investigated to explore the ion conductance in the presence of applied fields.

f) Applied-field MD simulations

Applied-field MD simulations were employed to examine the conductance of KCl through L and DpPorA as well as the L and DcWza pore. The required membrane potential across the OM was generated by applying an external homogeneous electric field E perpendicular to the membrane plane[10]. The external electric field acts on all charged particles of the system. The resulting membrane potential V generated is equal to the product of applied electric field E and the length of the simulation box in the applied-field direction L_z, i.e., $V = E \cdot L_z$.

The ionic currents have been estimated using[9]

$$I(t) = \frac{1}{L_z \Delta t} \sum_{i=1}^{N} q_i [z_i(t + \Delta t) - z_i(t)]. \qquad (2)$$

In this expression, q_i and z_i denote the charge and the *z* coordinate of atom *i*, whereas Δt describes the time step. For the simulations with 1 M KCl, we performed three 100 ns applied-field simulations for each pPorA and cWZa system at voltages of +0.2 +0.5 V, -0.2V, and -0.5V.

g) Structural insights of LcWza and DcWza pores

It should be noted that the flexible ends of the cWza pores occasionally showed unstable pore conformations in MD simulations resulting in partial disagreement between experimental and computational conductance values. The minimum average radii of LcWza and DcWza calculated using the HOLE program were 5.40 and 5.01 Å, respectively. Again, assuming the simplest model of a cylindrical pore, this difference suggests that LcWza should be about 1.16 times more conductive than DcWza **(Supplementary Fig. 19).** Moreover, the interior electrostatic potential maps for DcWza and LcWza are very similar. The C-terminal half of the pore possesses a negative electrostatic potential surface, whereas the N-terminal half of the pore shows a positive electrostatic potential in both pores **(Supplementary Fig. 19)**. It should be mentioned that the interior surface of the cWza pores consists of two acidic and two basic amino acid residues, which roughly neutralize the interior pore surface. Overall, the similarity

in the radii and electrostatic potentials for DcWza and LcWza are in accordance with the similar conductance values of these two pores.

h) Ramachandran Plot of the modeled pores

The converted D pores are accurate mirror pores of the L-pores. We then analyzed the sidechain reorientations due to the L to D-pore conversions and their effect on stability and ion conductivity. The structural changes induced by the chiral amino acids of the model pore were evaluated through the PROCHECK webserver[11]. An analysis of the Ramachandran plots suggests that 97.3 % of the amino acids are in the most allowed region, 2.3 % are in the allowed region, and no amino acid residue is in the disallowed region for the modeled LpPorA **(Supplementary Fig. 20)**. In the case of DpPorA, 42.8% of amino acids are in the most allowed region, 48 % in the allowed region, 9.0 in the additionally allowed region, with only 0.8% of the amino acid residues in the disallowed region. All the amino acid residues are in the left-handed helix region of the Ramachandran plot except two of the D-alanine residues, which reside in the disallowed region. For the LcWza pore, 98.7% of the amino acid residues are in the most allowed region and 1.3% in the allowed region. The Ramachandran plot analysis of DcWza demonstrated that 33.7%, 51.1%, and 10.3% are in the most allowed, allowed, and additionally allowed regions, respectively, whereas 1.3% are in the disallowed region. Three D-threonine residues reside in the disallowed region because of its two chiral centers[12]. The negative G-factor, a very good ERRAT score (above 95), and Verify 3D indicate a realistic model structure. It is to mention that a few crystal structures of D-amino acids containing protein, e.g., 3LQS and 1DAA, also contain 0.8% and 1.0% amino acids in the disallowed region.

Supplementary Tables:

**Nonaarginine added to the cis side**			
**Voltage [mV]**	**k_{on} [$M^{-1}s^{-1}$]** $\times 10^7$	**k_{off} [s^{-1}]** $\times 10^3$	**K_D [k_{off}/k_{on}] [M]** $\times 10^{-7}$
-10	153.8	2.27	14.76
-25	1.35	4	2963
-50	1.01	7.14	7069.3

Supplementary Table 1: Nonaarginine binding kinetics with DpPorA

The dissociation constants (K_D = k_{off}/k_{on}) were calculated from the association (k_{on}) and dissociation (k_{off}) rate constants. Electrolyte: 1 M KCl, 10 mM HEPES, pH 7.4. Nonaarginine was added to the cis side. Mean values (± s.d.) from at least three independent experiments (n = 3) are shown.

	-0.2V		+0.2V		
	G_{MD} (nS)	I_+/I_-	G_{MD} (nS)	I_+/I_-	G_{exp}(nS)
LpPorA	2.69±0.20	2.0±0.21	2.68±0.10	1.9±0.38	4.0
DpPorA	1.65±0.27	2.0±0.51	1.92±0.25	2.2±0.40	1.3
LcWza	1.49±0.08	2.7±0.23	1.54±0.10	2.0±0.44	0.75
DcWza	2.08±0.16	1.6±0.12	1.68±0.17	2.3±0.45	0.90

	-0.5V		+0.5V		
	G_{MD} (nS)	I_+/I_-	G_{MD} (nS)	I_+/I_-	G_{exp}(nS)
LpPorA	3.51±0.09	2.3±0.40	3.68±0.11	1.9±0.31	4.0
DpPorA	1.54±0.10	2.0±0.44	1.46±0.18	2.8±0.12	1.3
LcWza	1.11±0.07	2.8±0.46	1.89±0.08	1.7±0.12	0.75
DcWza	1.23±0.10	1.4±0.06	1.20±0.14	2.3±0.25	0.90

Supplementary Table 2: Average computed conductance values (GMD) for L and D pores Average computed conductance values (GMD) were obtained using applied-field MD simulations compared to the experimental counterparts (Gexp). Results for the voltage of ±0.2 V and ±0.5 V are given.

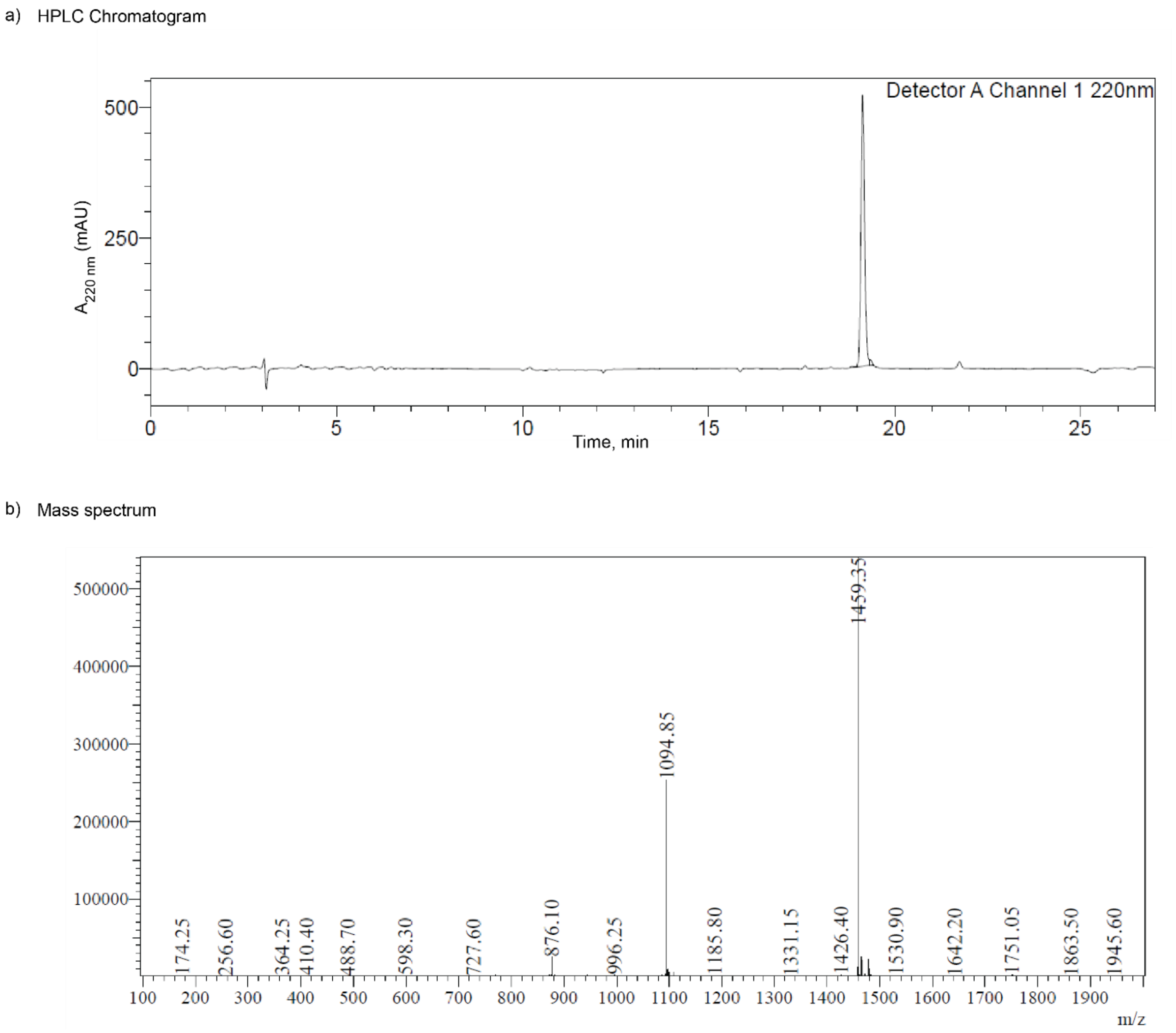

Supplementary Fig. 1: HPLC traces and mass spectra for DpPorA peptide.

a) HPLC traces (left, absorbance at 220 nm) and **b)** mass spectra (right) for DpPorA peptide. Calculated mass = 4376.134 Da, observed mass = 4376.2 Da.

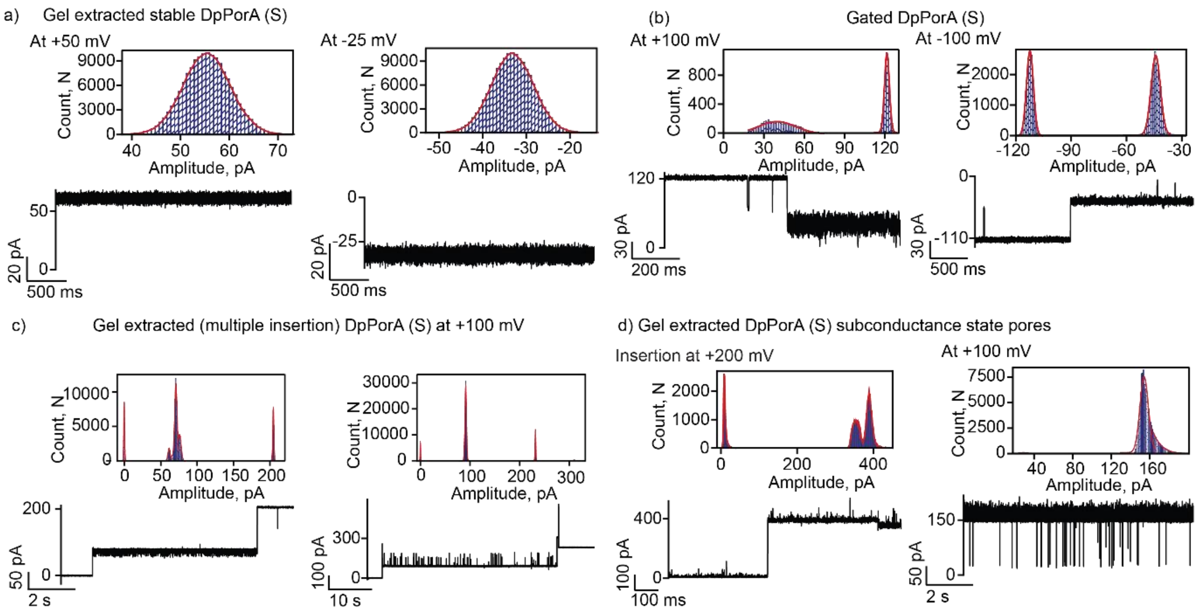

Supplementary Fig. 2: Electrical recordings of gel extracted DpPorA (S).

a) DpPorA (gel extract) stable states at +50 mV and -25 mV. **b)** Electrical recording of gel extracted DpPorA (gel extract) showing gating at +100 mV and -100 mV. **c)** Electrical recording of multiple insertions of gel extracted DpPorA into a planar bilayer at +100 mV. **d)** Electrical recording of gel extracted DpPorA showing subconductance states at +200 mV and +100 mV. Corresponding all-points histograms are shown in the inset. The current signals were filtered at 2 kHz and sampled at 10 kHz. Electrolyte: 1 M KCl, 10 mM HEPES, pH 7.4.

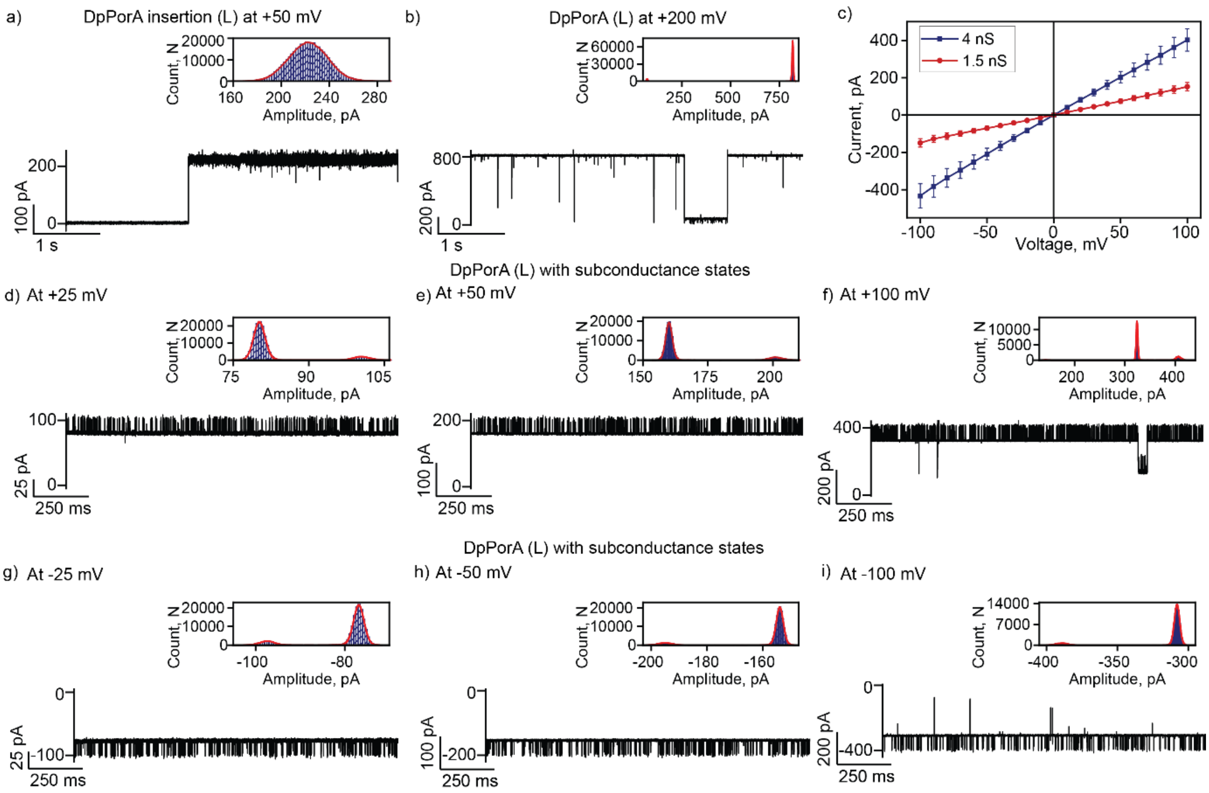

Supplementary Fig. 3: Electrical recordings of gel extracted DpPorA (L).

a) Single DpPorA (gel extract) insertion at +50 mV **b)** Electrical recording of single DpPorA showing steady conductance at +200 mV. **c)** I–V curve comparison of two different single DpPorA pores (S and L states). Error bars represent 15% standard error mean between four independent single channel experiments. **d)** Electrical recording of single DpPorA showing sub conductance states at +25 mV **e)** at +50 mV and **f)** at +100 mV. **g)** Electrical recording of single DpPorA showing sub conductance states at -25 mV **h)** at -50 mV and **i)** at -100 mV. The current signals were filtered at 2 kHz and sampled at 10 kHz. Electrolyte: 1 M KCl, 10 mM HEPES, pH 7.4.

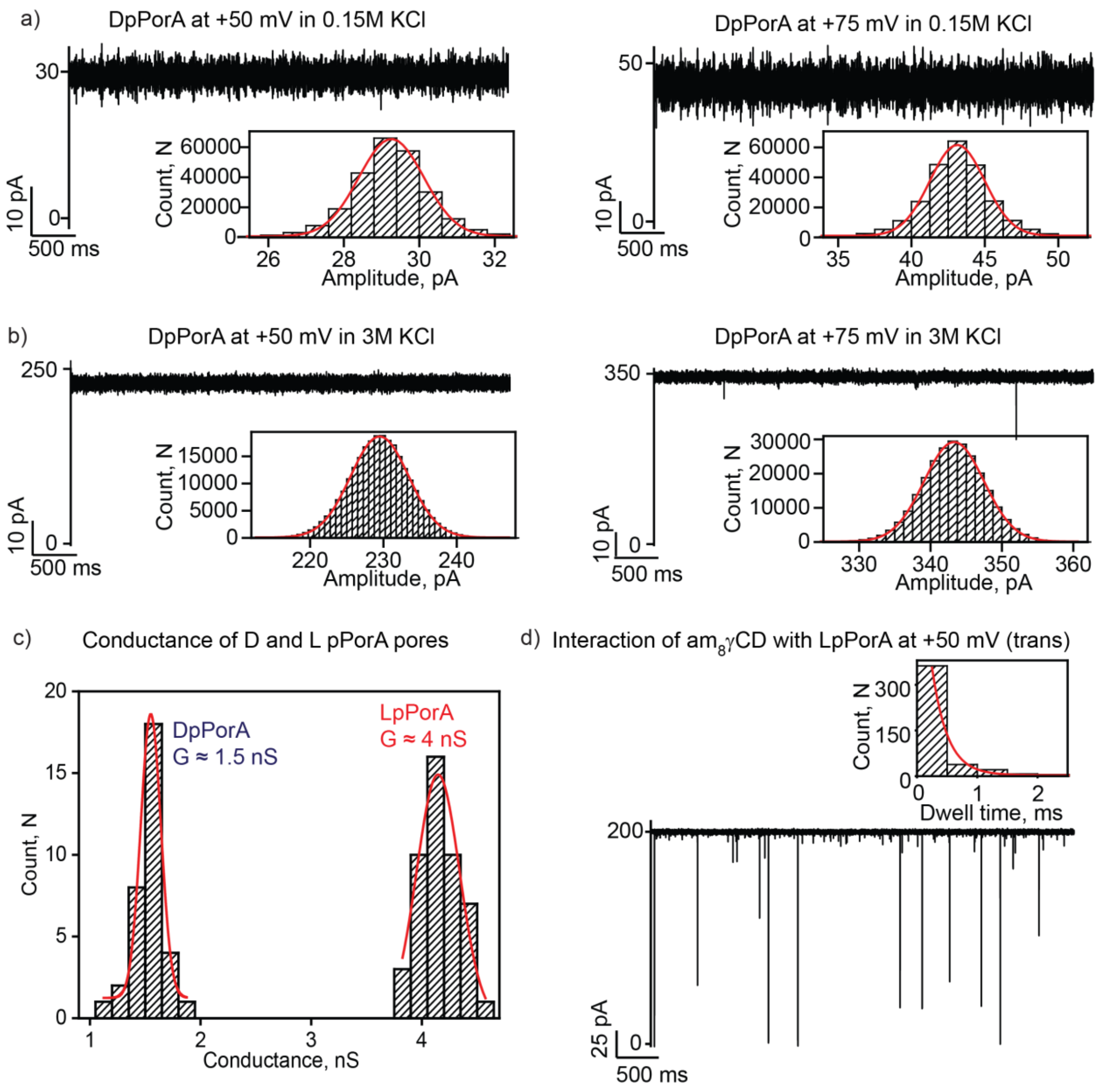

Supplementary Fig. 4: Electrical properties of DpPorA at different salt conditions and comparison with LpPorA.

a) Electrical recording of DpPorA at low salt buffer (0.15 M KCl, 10 mM HEPES, pH 7.4). **b)** Electrical recording of DpPorA at high salt buffer (3 M KCl, 10 mM HEPES, pH 7.4). **c)** Unitary conductance histograms of D and LpPorA at +50 mV by fitting the distribution to a Gaussian. **d)** Interaction of am$_8$γCD (1 µM, trans) with LpPorA at + 50 mV. Electrolyte: 1 M KCl, 10 mM HEPES, pH 7.4. The current signals were filtered at 2 kHz and sampled at 10 kHz.

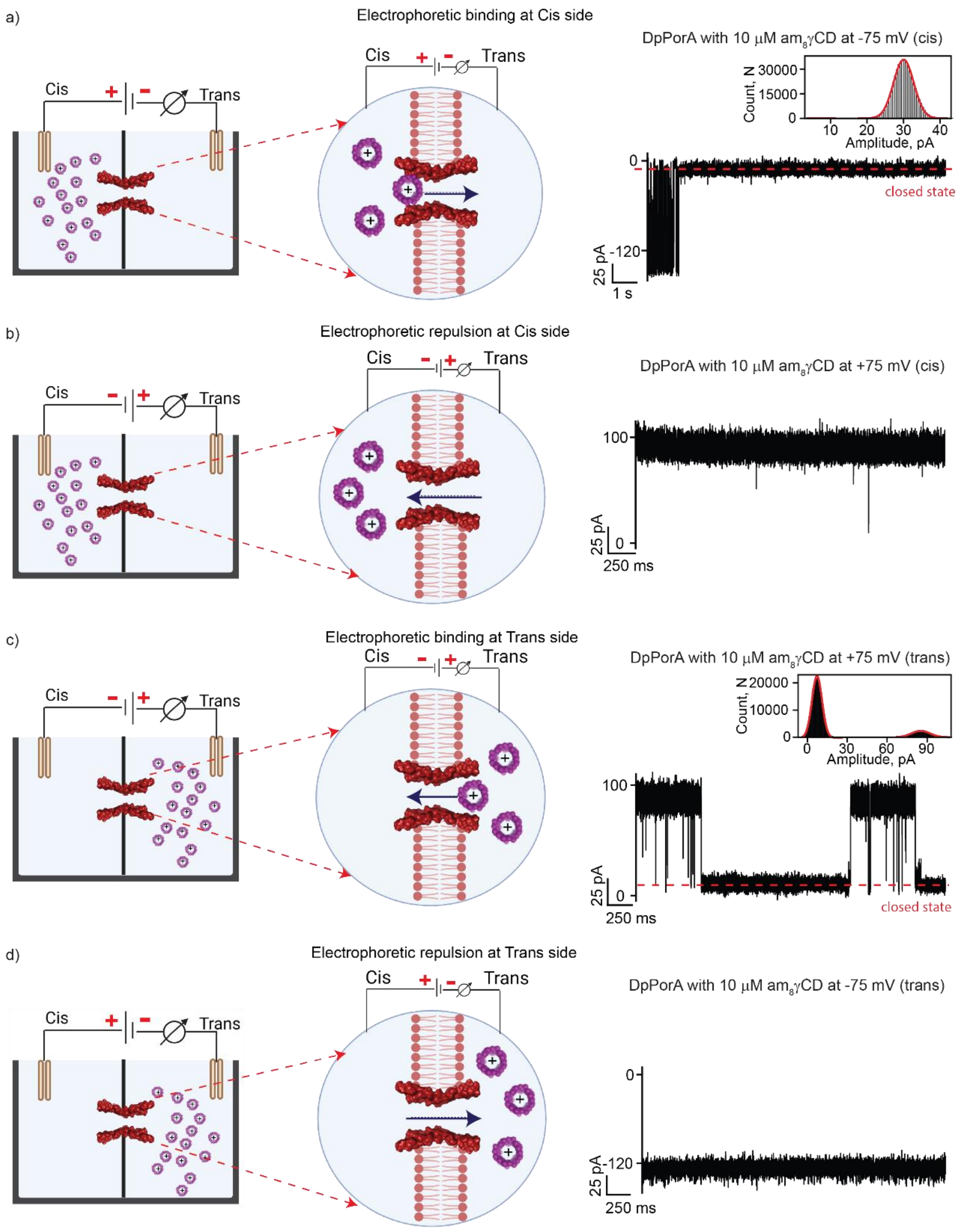

Supplementary Fig. 5: Interaction of DpPorA with cationic am8γCD

a) Schematic and voltage-driven translocation of am8γCD through DpPorA (10 µM, cis) at -75 mV. **b)** Schematic and electrophoretic repulsion of am8γCD from DpPorA (10 µM, cis) at +75 mV **c)** Schematic and voltage-driven translocation of am8γCD through DpPorA (10 µM, trans) at +75 mV. **d)** Schematic and electrophoretic repulsion of am8γCD from DpPorA (10 µM, trans) at -75 mV. The inset shows the corresponding current-amplitude histogram. The current signals were digitally filtered at 2 kHz. The schematics are created with BioRender.com.

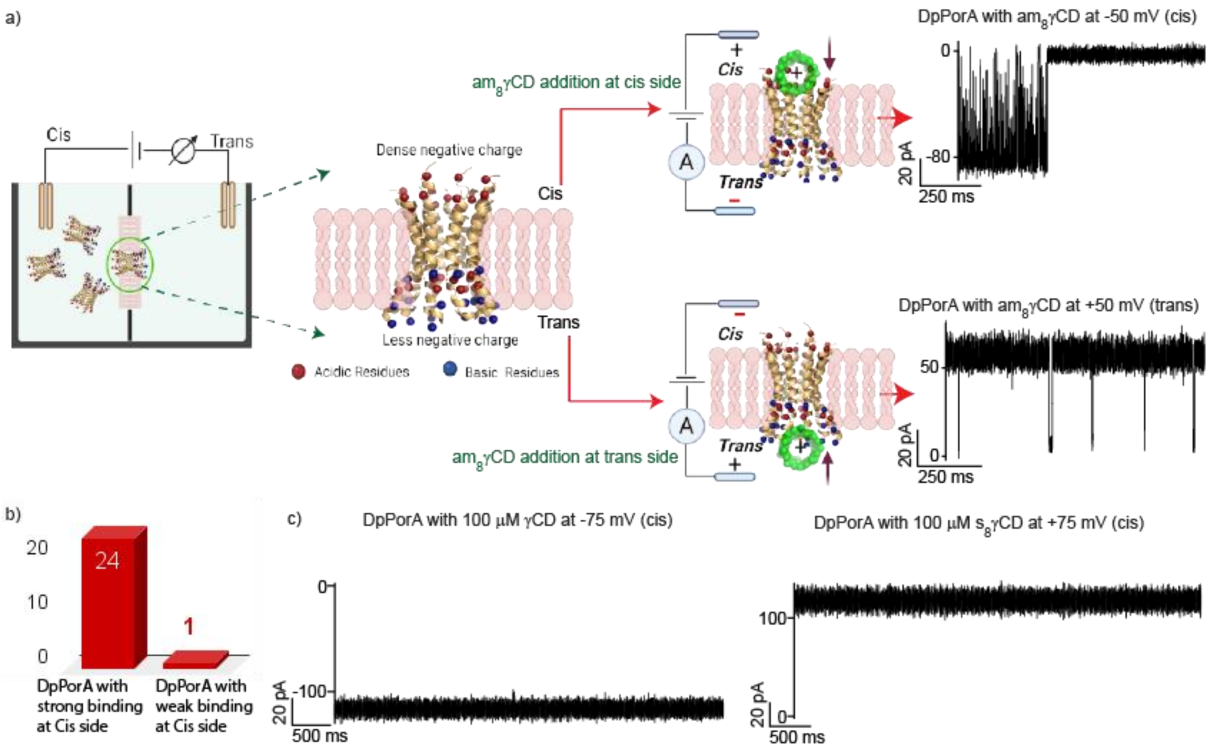

Supplementary Fig. 6: Interaction of DpPorA with differently charged cyclic sugars and PEG polymers.

a) Asymmetry in the cationic cyclic sugar binding with DpPorA and schematic showing orientation of DpPorA in lipid bilayers based on the cationic am$_8$γCD binding. **b)** Bar graph representing the pore orientation based on am$_8$γCD binding. **c)** Interaction of neutral γCD with single DpPorA (100 µM, cis) at -75 mV and interaction of anionic s$_8$γCD with single DpPorA (100 µM, cis) at +75 mV. The current signals were digitally filtered at 2 kHz. The schematics are created with BioRender.com.

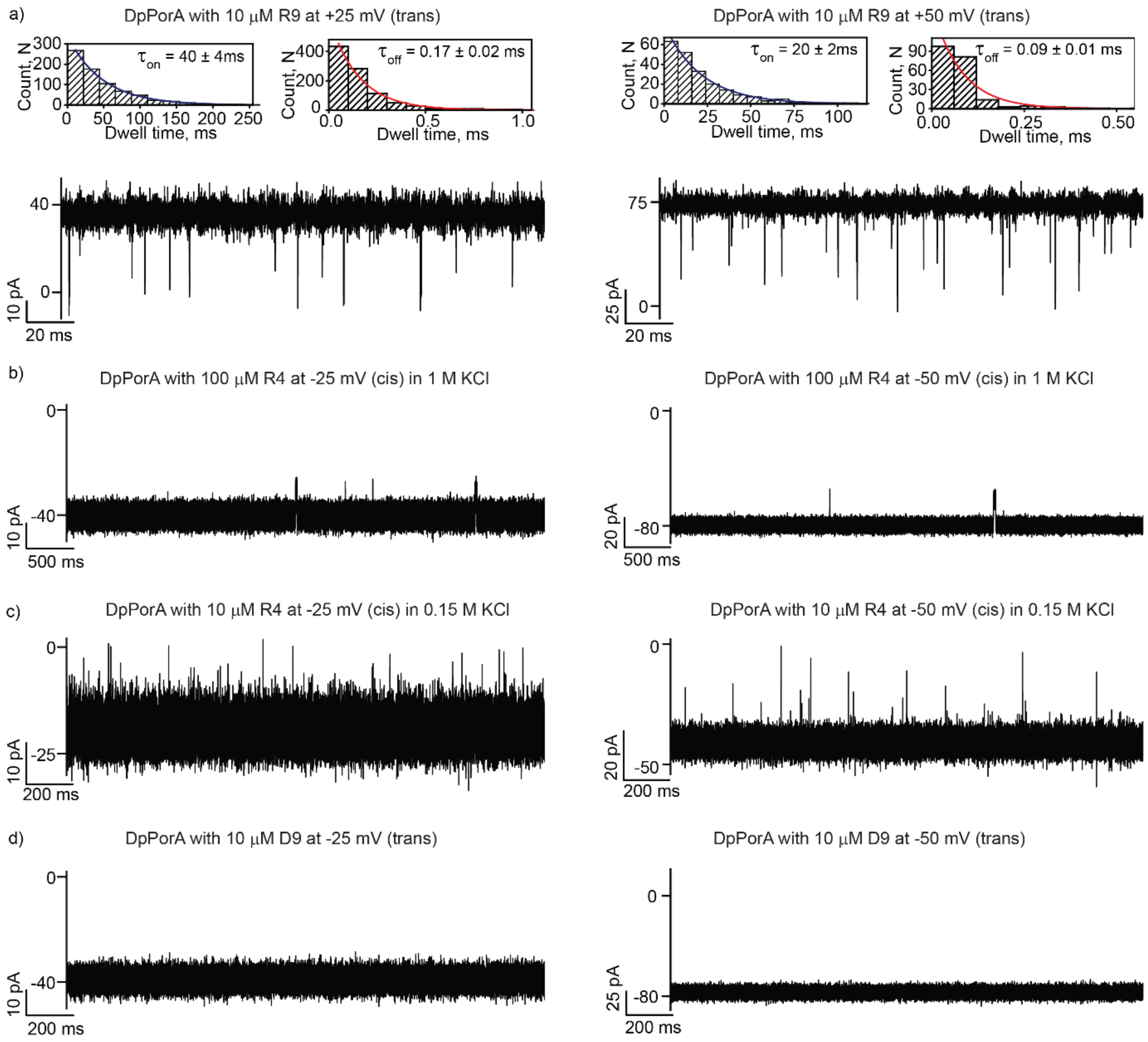

Supplementary Fig. 7: Interaction of DpPorA with differently charged peptides

a) Interaction of nonoarginine (R9) with single DpPorA (10 µM, trans) at +25 mV and +50 mV. The corresponding τ_{off} and τ_{on} dwell time histogram of R9 blocking fitted with a monoexponential probability function is shown. **b)** Interaction of tetraaarginine with single DpPorA (10 µM, cis) at -25 mV and -50 mV at high salt buffer. **c)** Interaction of tetraaarginine with single DpPorA (10 µM, cis) at -25 mV and -50 mV at low salt buffer. **d)** Interaction of nonaaspartate with single DpPorA (10 µM, trans) at -25 mV and -50 mV. The current signals were digitally filtered at 7 kHz.

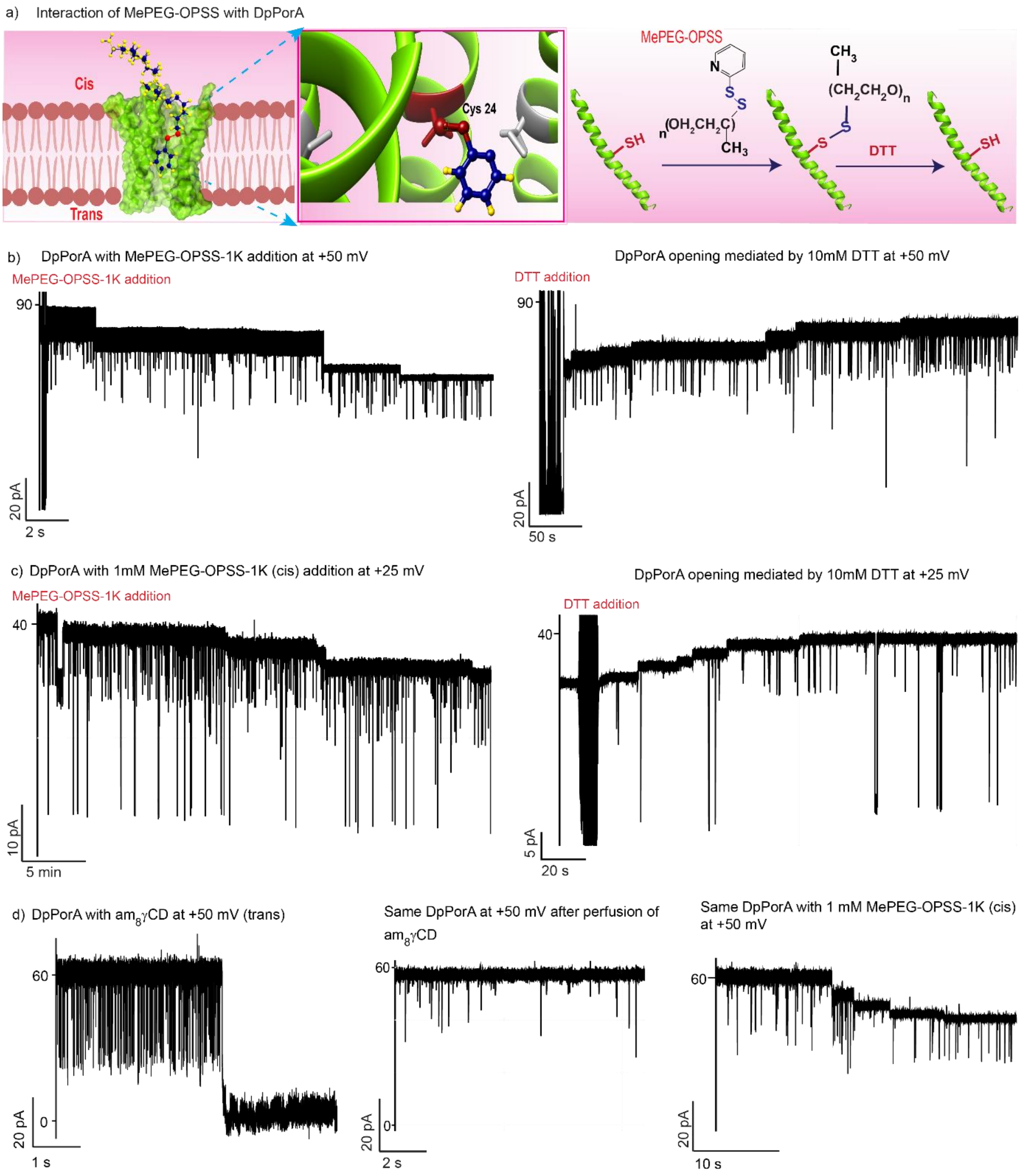

Supplementary Fig. 8: Interaction of DpPorA with PEG polymers

a-c) Schematic and electrical recordings (two independent experiments) showing the reversible chemical modification of 1 mM MePEG-OPSS-1k with DpPorA at +50 mV and +25 mV and the addition of 10 mM DTT resulted in the pore opening. **d)** Electrical recordings showing the interaction of am₈γCD with DpPorA (10 µM, trans) at +50 mV and chemical modification of 1 mM MePEG-OPSS-1k with the same DpPorA at +50 mV after perfusion of CDs. The current signals of the PEG experiments were digitally filtered at 500 Hz and am₈γCD interaction was filtered at 2 kHz.

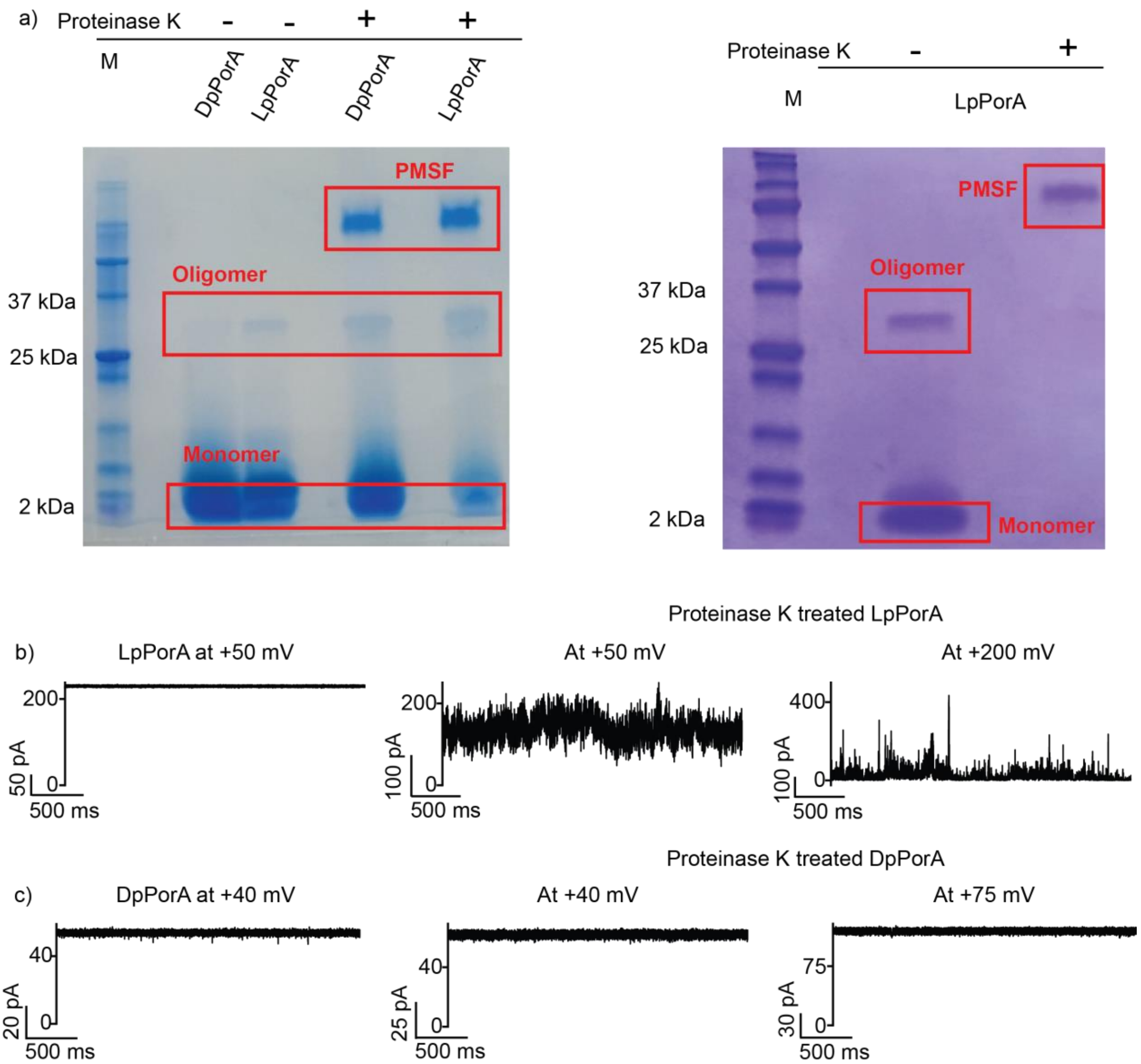

Supplementary Fig. 9: Functional stability of DpPorA and LpPorA to protease reaction.

a) DpPorA and LpPorA peptides treated and untreated with proteinase K upon SDS-polyacrylamide gel electrophoresis alongside denatured protein standards. Data are representative of more than three repeats. **b)** Electrical recording of LpPorA in the absence and presence of proteinase K at different voltages. **c)** Electrical recording of DpPorA in the absence and presence of proteinase K at different voltages. The current signals were filtered at 2 kHz and sampled at 10 kHz.

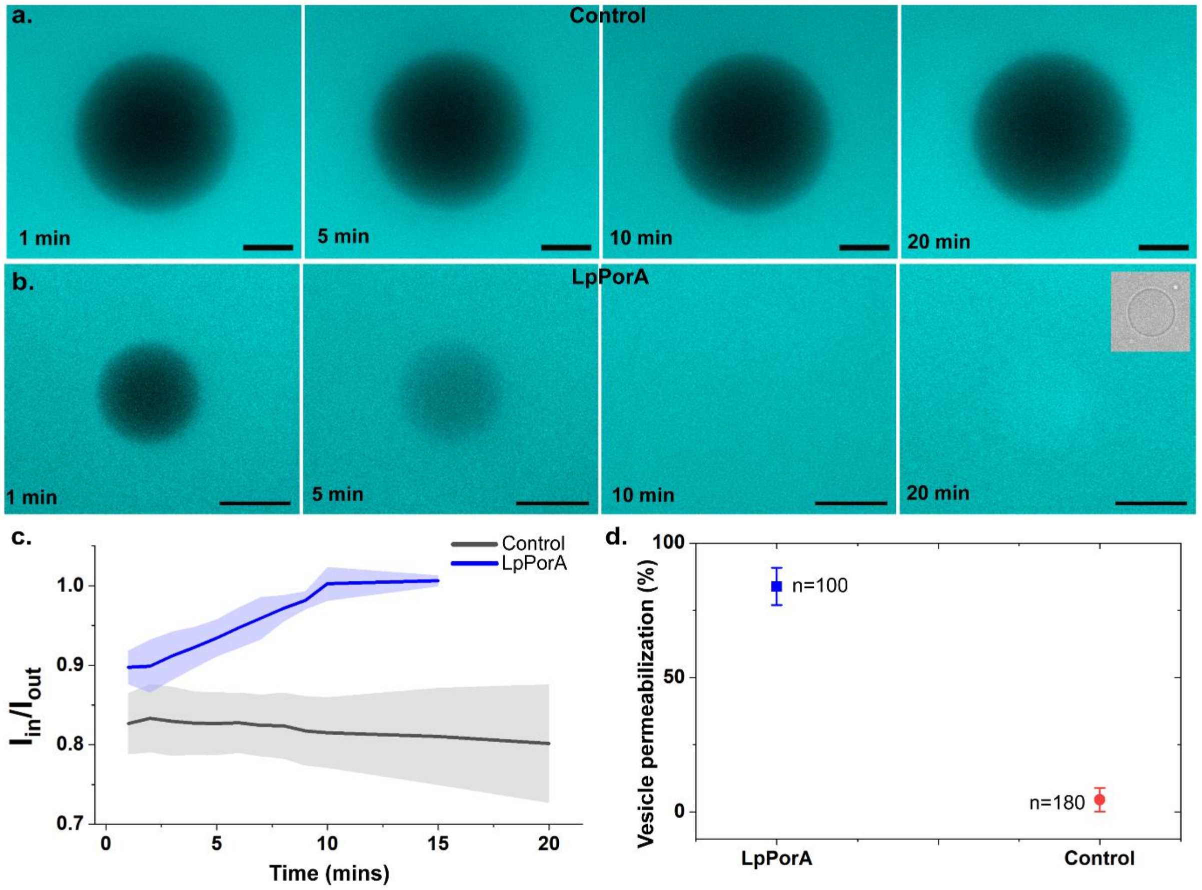

Supplementary Fig. 10: Functional assembly of LpPorA in giant vesicles

Representative fluorescence image of single vesicle displaying time-dependent uptake of dye **a)** in the absence of peptides **b)** in the presence of LpPorA peptides, where false blue color represents the Alexa-Fluor 350 dye in vesicles. Inset shows the phase-contrast image of the vesicle. **c)** Time-dependent curve of normalized intensity of single vesicles with LpPorA (n=13 individual vesicles) and without peptide (n=10 individual vesicles). Blue and black lines represent the mean normalized intensity over time and the shaded region (error bands) represents the standard error of the mean. **d)** Vesicle permeabilization percentage is shown in the absence and presence of LpPorA. Permeabilization percentage of LpPorA is 83 ± 6.9% (blue, mean ± SD from n = 100 vesicles and N= 5 independent batches) and control is 4.5 ± 4.4% (red, mean ± SD from n = 180 vesicles and N= 5 independent batches). Buffer conditions: 100 mM KCl, 10 mM HEPES pH 7; scale bar: 10 µm.

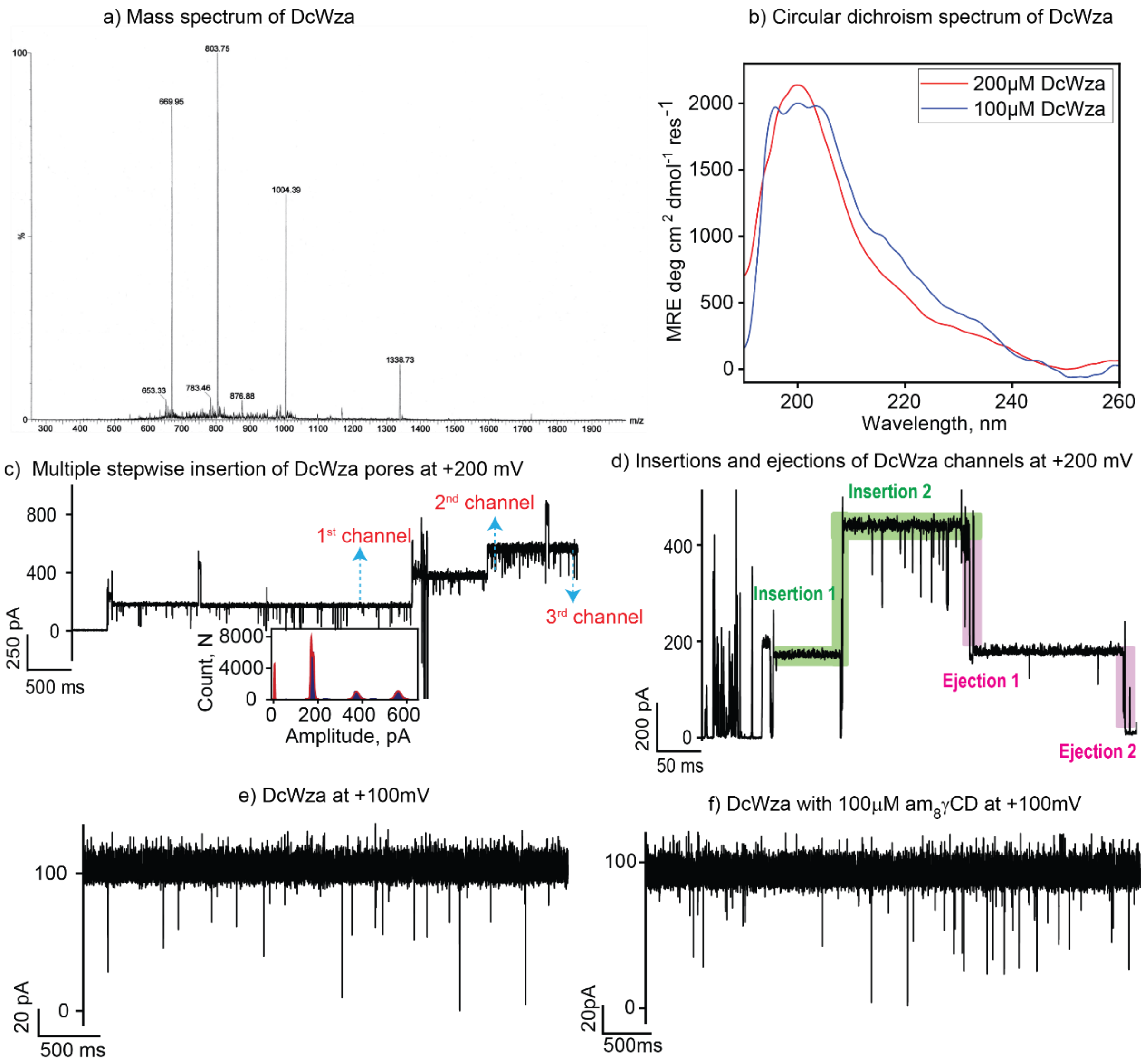

Supplementary Fig. 11: Biophysical and electrical properties of transient DcWza.

a) Mass spectrum of DcWza peptides. DcWza peptide calculated mass = 4014.684 Da, observed mass = 4014.7120. **b)** The CD spectra for 200 µM and 100 µM DcWza peptides in phosphate-buffered saline with 1% DDM**. c)** Electrical recording of multiple insertions of DcWza at +200 mV. **d)** Two rapid single DcWza insertion events and ejections at +200 mV. **e-f)** Electrical recording of transient DcWza that remained in the open conductance state in the absence and presence of 100 µM (trans) $am_8\gamma CD$ at +100 mV. The current signals were filtered at 2 kHz and sampled at 10 kHz. Electrolyte: 1 M KCl, 10 mM HEPES, pH 7.4.

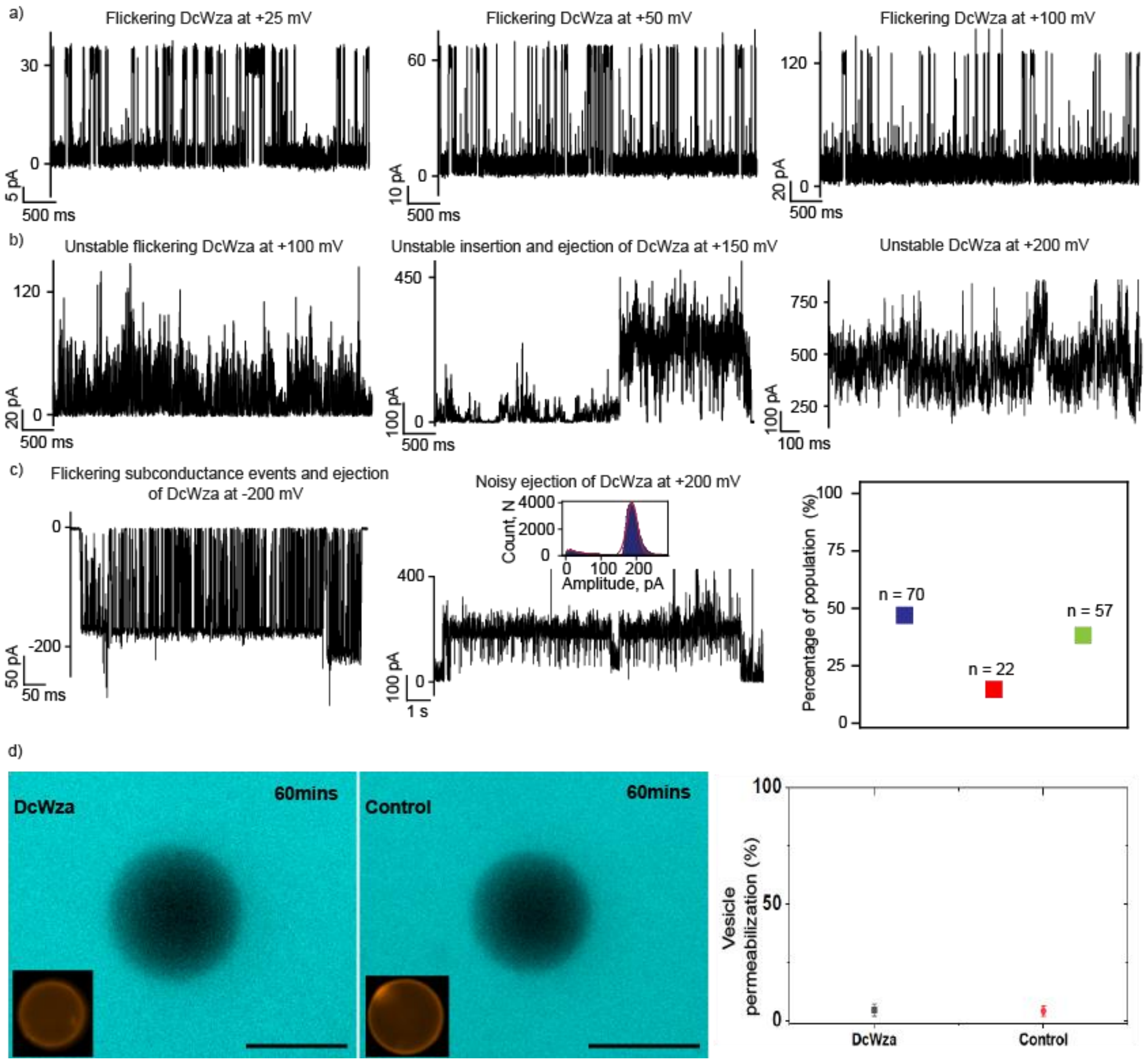

Supplementary Fig. 12: Electrical properties of DcWza and transport in giant vesicle system **a)** Electrical recording of DcWza showing flickering current states at +25 mV, +50 mV, +100 mV and **b)** unstable noisy DcWza at +100 mV, +150 mV and +200 mV **c)** Insertion and ejection of DcWza at ±200 mV. Plot representing different DcWza populations (stable in blue, unstable in red and closed in green). Electrolyte: 1 M KCl, 10 mM HEPES, pH 7.4. The current signals were filtered at 2 kHz. **d)** Fluorescence image of a single vesicle revealing no dye transport at 60 min in the presence and absence of DcWza, where false blue color represents the Alexa-Fluor 350 dye in vesicles. Inset shows the vesicle fluorescently labeled with 0.05 mol% ATTO-550 DOPE. Permeabilization percentage of DcWza is 4.6 ± 2.5% (Black, mean ± SD from n = 100 vesicles and N= 3 independent batches) and control is 4.2 ± 2.1% (Red, mean ± SD from n = 90 vesicles and N= 3 independent batches) Buffer conditions: 100 mM KCl, 10 mM HEPES pH 7, Scale bar: 10 µm.

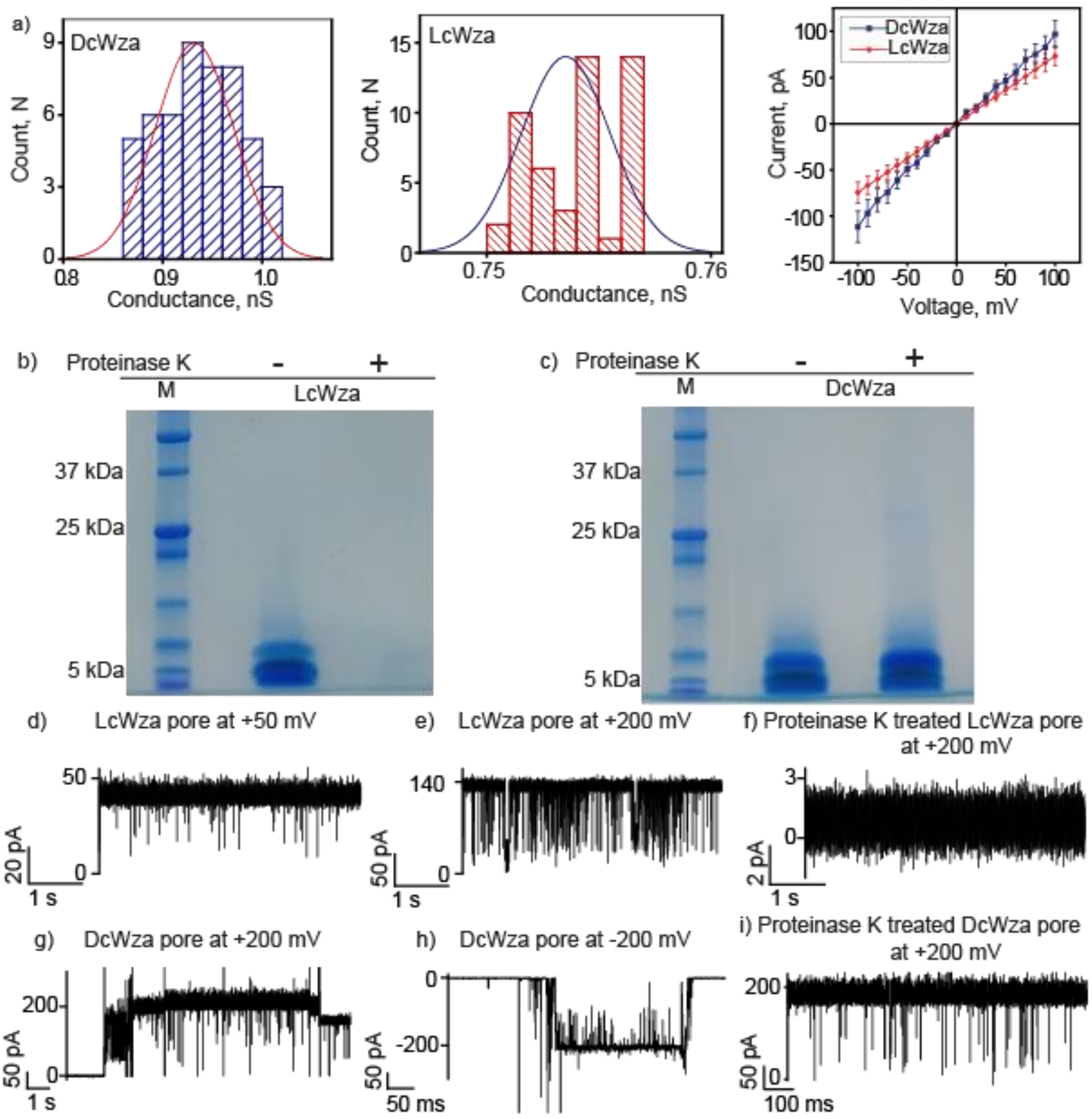

Supplementary Fig. 13: Comparison of the electrical properties of D and LcWza and functional stability of D and LcWza to Proteinase K

a) The unitary conductance histograms of DcWza and LcWza by fitting the distribution to a Gaussian and I−V curve obtained from a single DcWza and LcWza. Error bars represent 15% standard error mean between 3 independent experiments. **b-c)** DcWza peptides run on SDS-PAGE. Proteinase K treated and untreated LcWza and DcWza peptides. Data are representative of more than three repeats. **d-e)** Electrical recording of LcWza in the absence of proteinase K at +50 mV and +200 mV. **f)** Electrical recording of LcWza in the presence of proteinase K at +200 mV. **g-h)** Electrical recording of DcWza in the absence of proteinase K at ± 200 mV. **i)** Electrical recording of DcWza in the presence of proteinase K at +200 mV. The current signals were filtered at 2 kHz and sampled at 10 kHz. Electrolyte: 1 M KCl, 10 mM HEPES, pH 7.4.

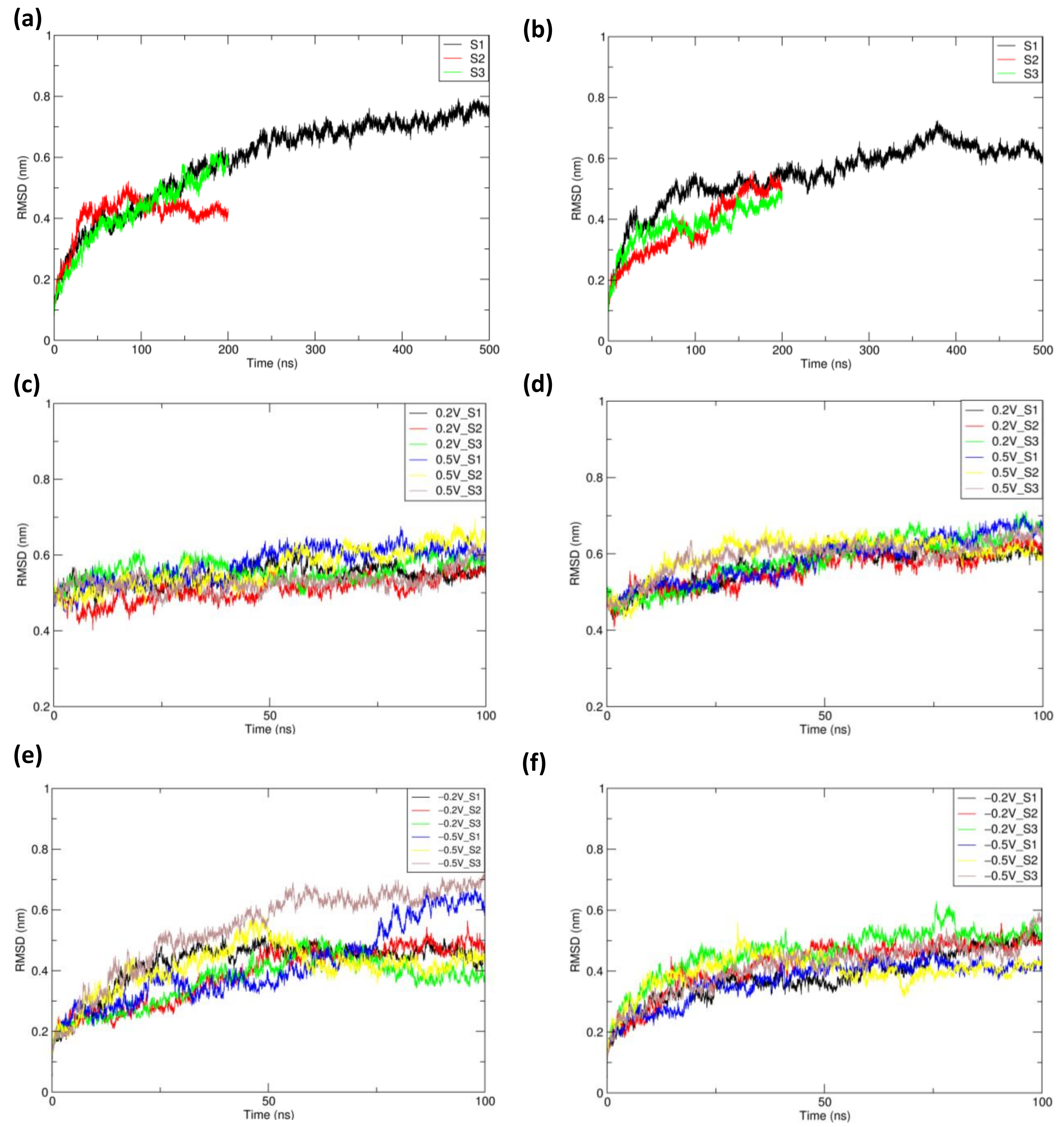

Supplementary Fig. 14: Protein backbone-backbone RMSD values with respect to the initial pPorA structures for 200 ns unbiased MD simulations.

a) LpPorA and **b)** DpPorA unbiased simulation. The structural deviations of pPorA due to the applied-field MD simulations were examined by RMSD calculations at the end of the 200 ns unbiased simulations. **c)** LpPorA and **d)** DpPorA applied-field simulation at +0.2V and +0.5V. **e)** LpPorA and **f)** DpPorA applied-field simulation at -0.2V and -0.5V. S1, S2, and S3 denote the three individual simulations. One of the simulations was extended to 500 ns.

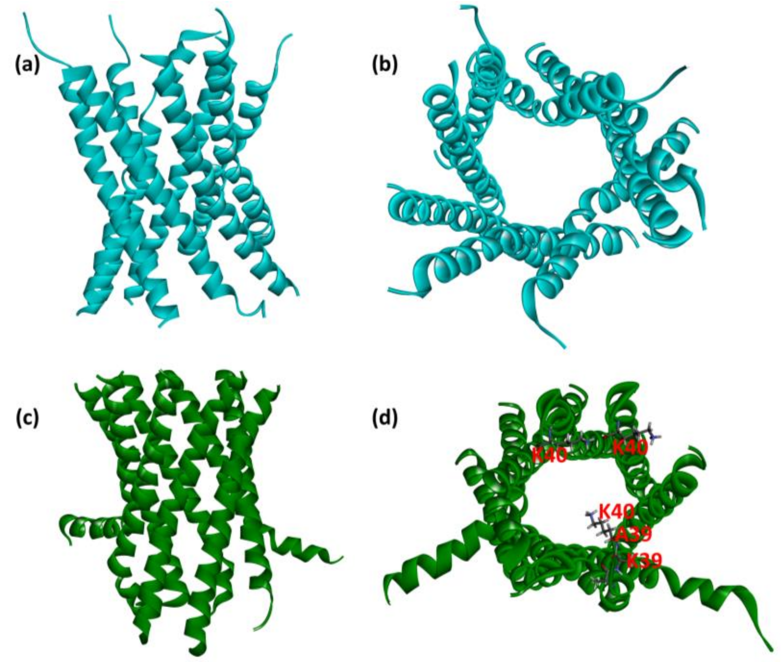

Supplementary Fig. 15: Final pPorA conformations

Final pPorA conformations at + 0.5 V applied voltage **(a, b)** LpPorA. **(c, d)** DpPorA.

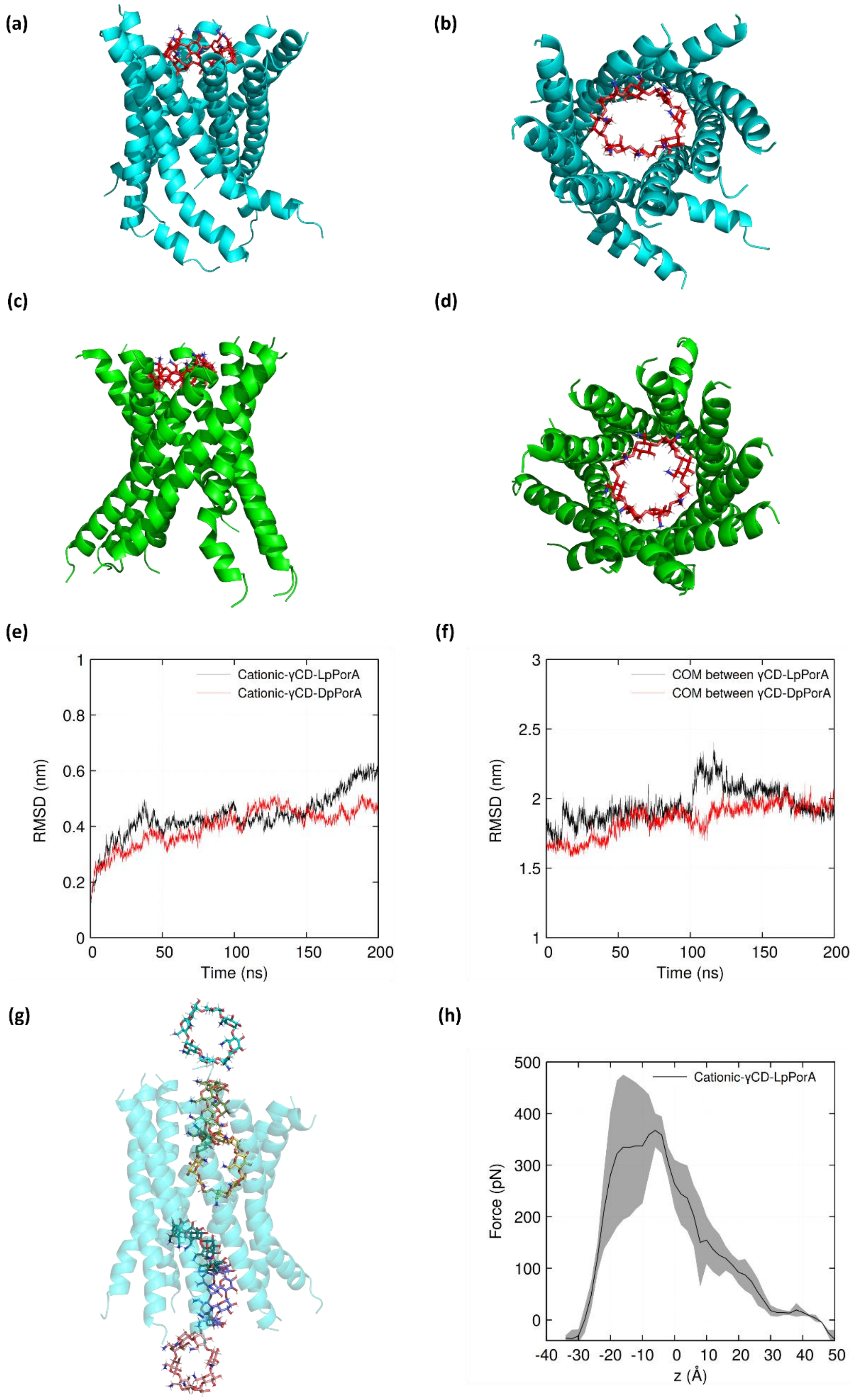

Supplementary Fig. 16: Cationic am$_8\gamma$CD bound to L and DpPorA.

(a-b) Binding of am$_8\gamma$CD with LpPorA. **(c-d)** Binding of am$_8\gamma$CD with DpPorA. The cationic molecule strongly interacts with the aspartic acid via hydrogen bonding and electrostatic interactions. **e)** The RMSD of pore and molecule together was calculated with respect to that initial structure. **f)** The center of the mass distance between pPorA and am$_8\gamma$CD. **g)** Transport of am$_8\gamma$CD through LpPorA. **h)** Average force profile from steered MD simulation for the permeation of am$_8\gamma$CD as the function of reaction coordinate z, i.e., the COM distance between am$_8\gamma$CD and the C_α atoms of LpPorA and the shaded error bars represent the standard deviations.

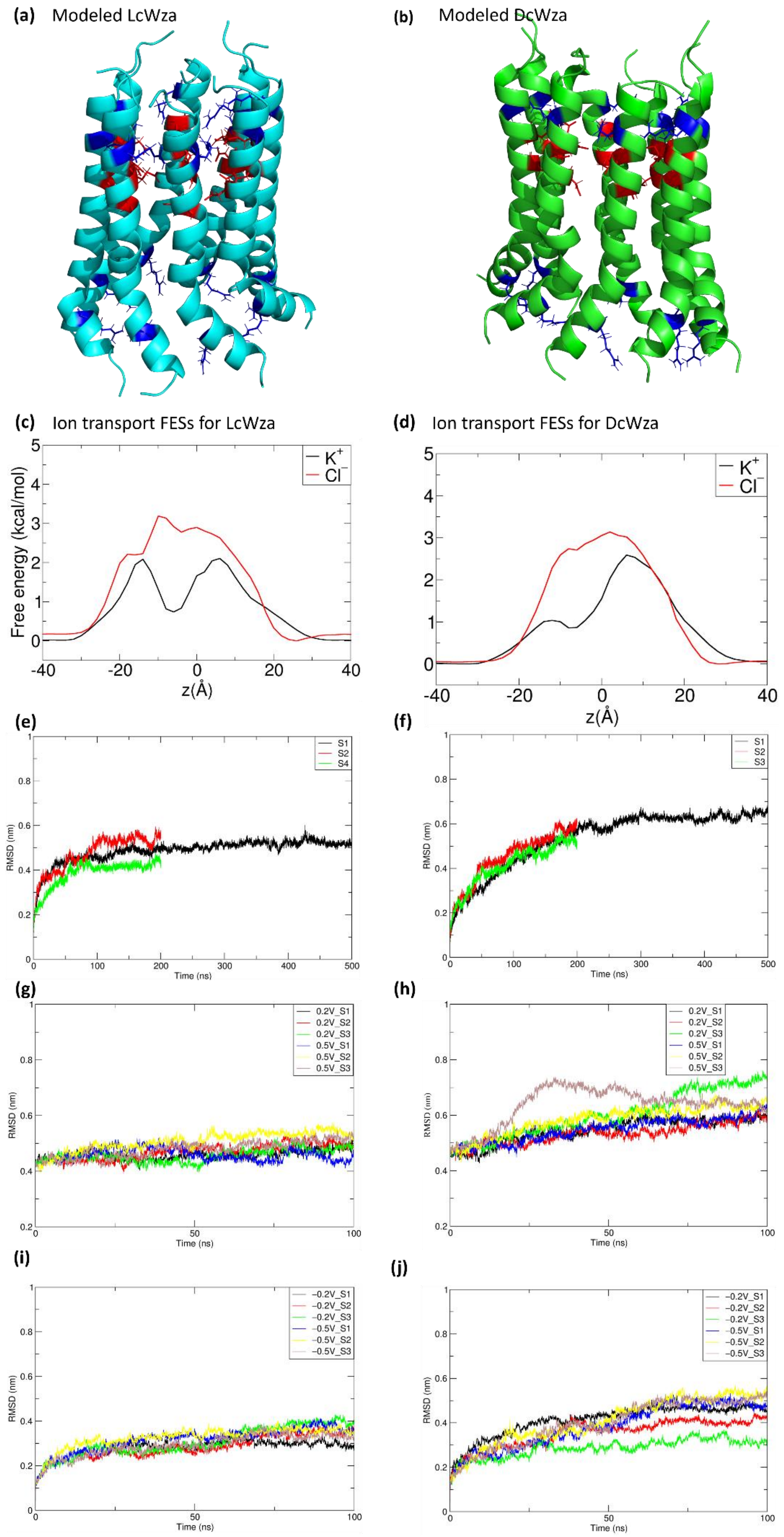
(a) Modeled LcWza
(b) Modeled DcWza
(c) Ion transport FESs for LcWza
(d) Ion transport FESs for DcWza
Free energy (kcal/mol)
z(Å)
K+
Cl−
(e)
(f)
S1
S2
S4
S3
RMSD (nm)
Time (ns)
(g)
(h)
0.2V_S1
0.2V_S2
0.2V_S3
0.5V_S1
0.5V_S2
0.5V_S3
(i)
(j)
−0.2V_S1
−0.2V_S2
−0.2V_S3
−0.5V_S1
−0.5V_S2
−0.5V_S3

Supplementary Fig. 17: Protein backbone-backbone RMSD values with respect to the initial cWza structures for 200 ns unbiased MD simulations.

a) Structures of the designed LcWza pore after 200-ns-long unbiased MD simulations. **b)** DcWza stable structure in cartoon representation. **c)** 1D Multi-ion free energy profiles for transport of K^+ and Cl^- ions along the LcWza channel axis and **d)** Multi-ion free energy profiles for K^+ and Cl^- ions transport through DcWza. **e)** LcWza unbiased simulations. **f)** DcWza unbiased simulations. **g)** LcWza applied-field simulation at +0.2V and +0.5V. **h)** DcWza applied-field simulation at +0.2V and +0.5V. **i)** LcWza applied-field simulation at -0.2V and -0.5V. **j**) DcWza applied-field simulation at -0.2V and -0.5V.

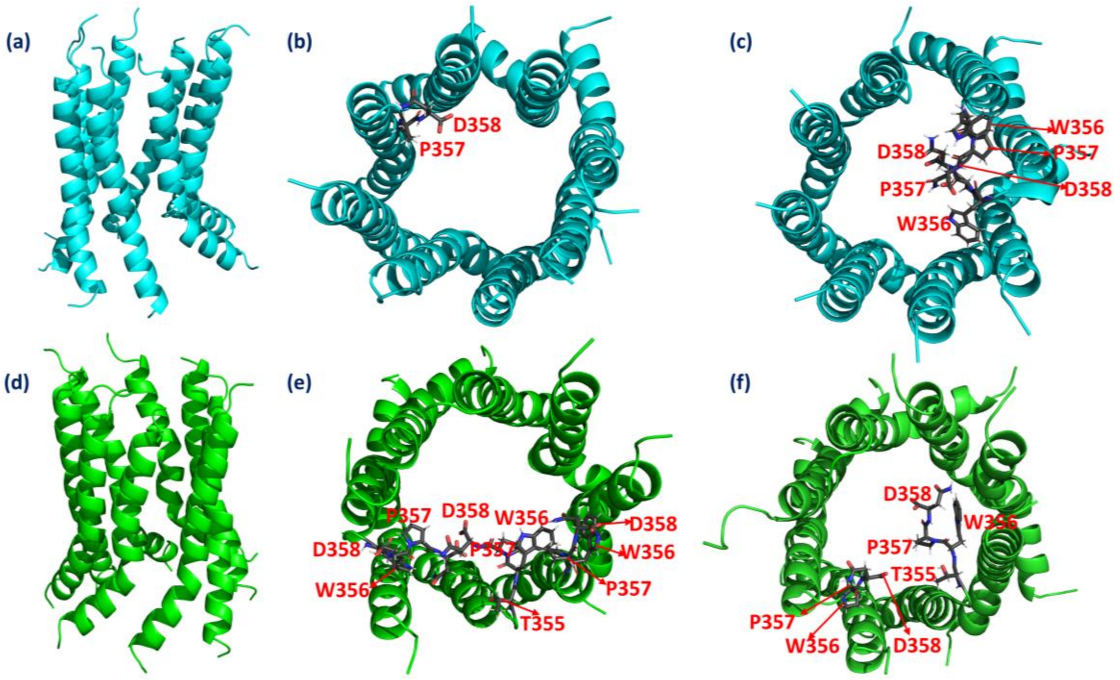

Supplementary Fig. 18: Final L and DcWza conformations.

Final L and DcWza conformations after the -0.5V and +0.5V applied-field simulations. The C-terminal asparagine, proline, and tryptophan amino acid residues are prone to form coil structures that hinder ion transport. **(a, b)** LcWza at +0.5V **c)** LcWza at -0.5V **(d, e)** DcWza at +0.5V. **f)** LcWza at -0.5V.

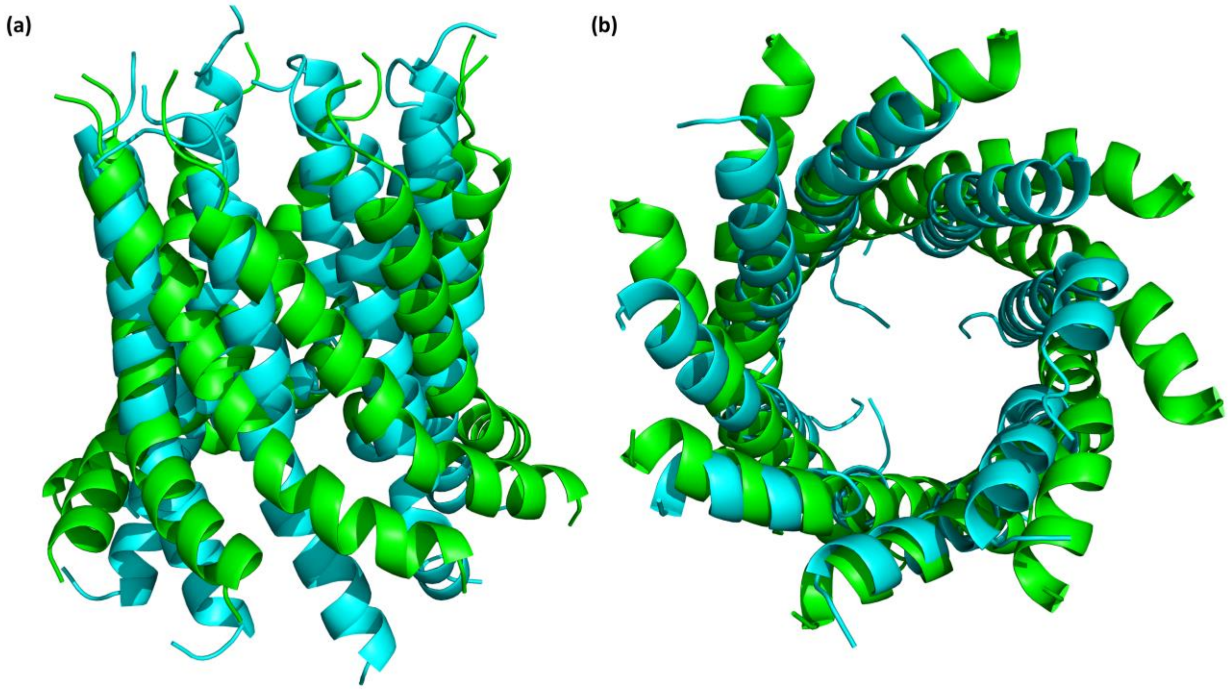

Supplementary Fig. 19: Crystal structure of the α-helical outer membrane D4 domain of Wza compared to the designed cWza structure.

a) The crystal structure, PDB ID: 2J58, of Wza (green), is reported, and **b)** modeled the cWza protein (cyan) extracted from the Wza crystal structure (after a 200 ns-long unbiased MD simulation). Structural analysis suggests that the modeled structure is narrower than the crystal structure in the C- and N-terminal domain regions, although the pore diameter remains approximately 17Å.

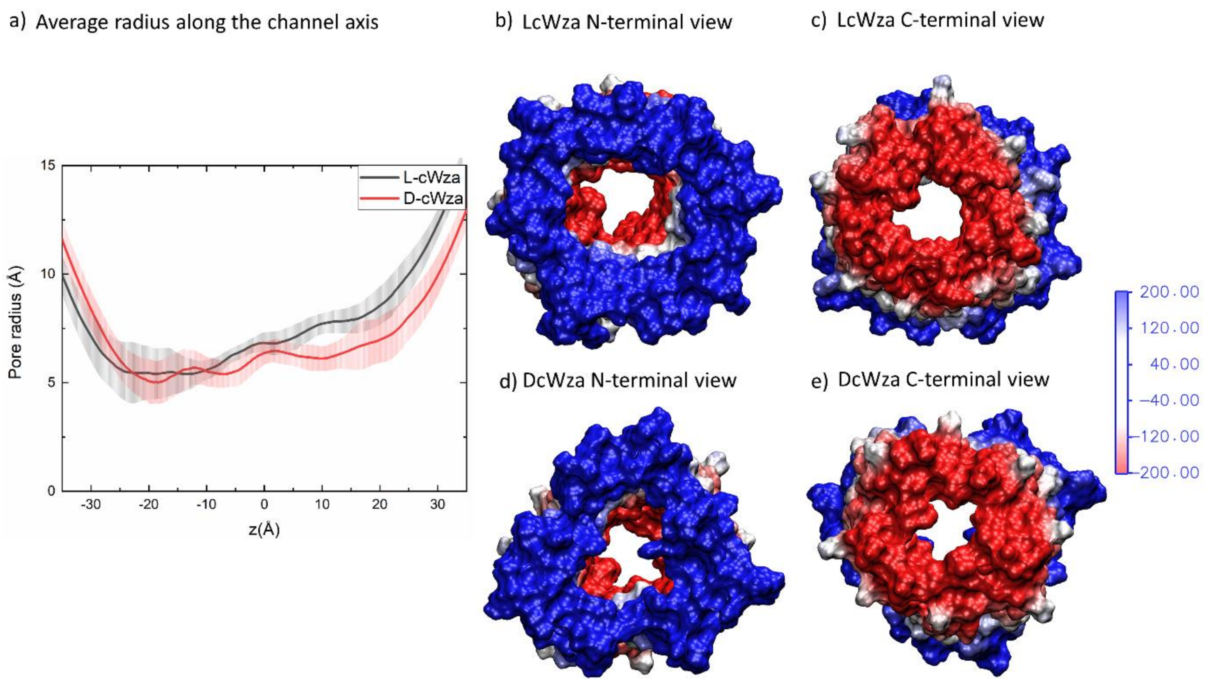

Supplementary Fig. 20: Radius along the channel axis and electrostatic potential of the DcWza and LcWza pores.

a) Radius profile along the channel axis averaged over a trajectory also showing the standard deviations as the shaded error bars. **b, c)** N-terminal and C-terminal view of the electrostatic potential map of the amino residues of LcWza lining the interior channel wall. **d, e)** N-terminal and C-terminal view of the electrostatic potential map of the amino residues of DcWza lining the interior channel wall. The calculated electrostatic potentials vary from -695 to +600 k_BT/e for LcWza and from -674 to +683 k_BT/e for DcWza.

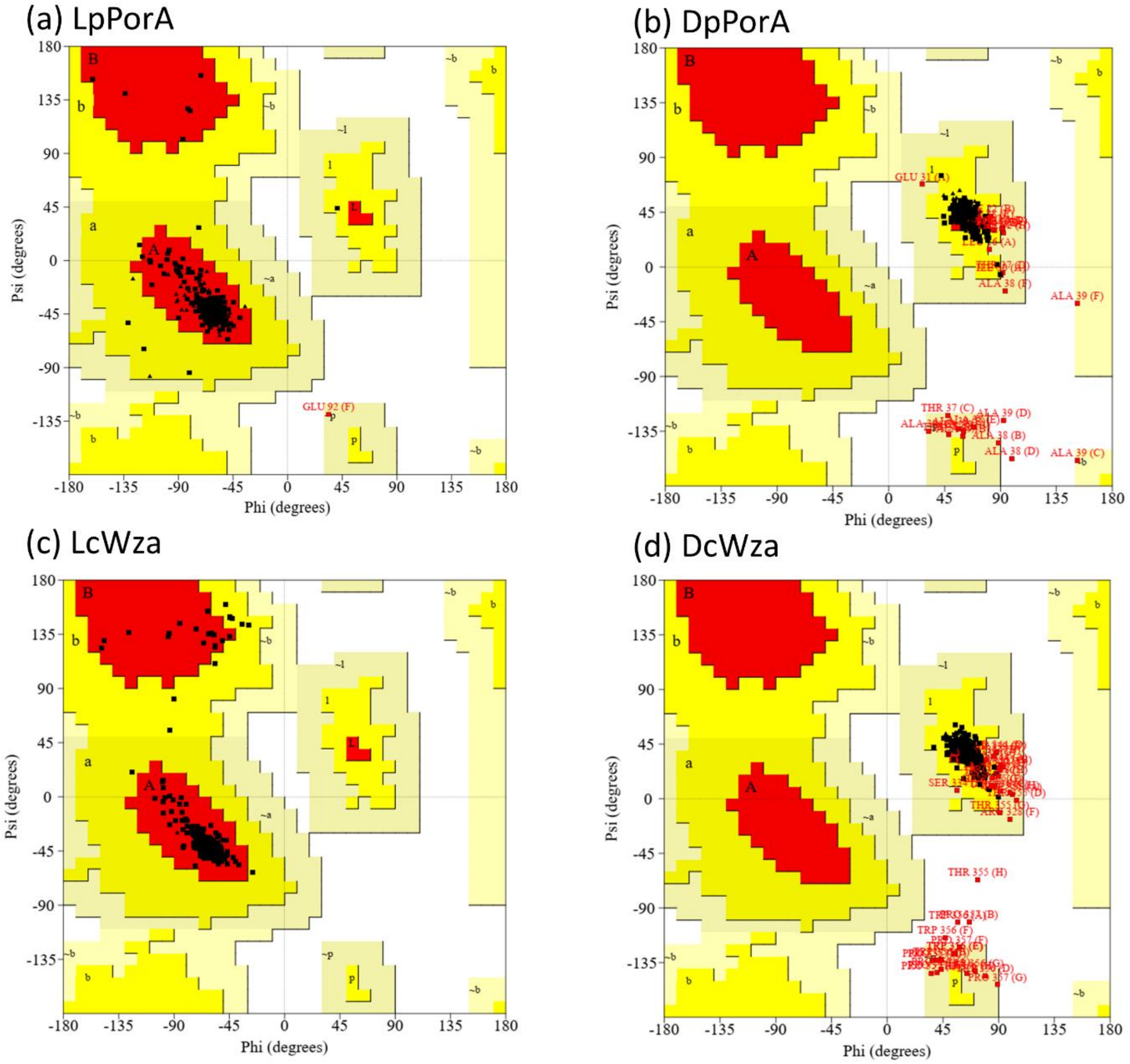

Supplementary Fig. 21: Ramachandran plots of the modeled pores.

a) Ramachandran plot of LpPorA calculated from the PROCHECK webserver. **b)** Ramachandran plot of DpPorA. **c)** Ramachandran plot of LcWza. **d)** Ramachandran plot of DcWza.

Supplementary References

1. Benz, R., Schmid, A. & Hancock, R.E. Ion selectivity of gram-negative bacterial porins. J. Bacteriol. 162, 722-727 (1985).

2. Cheley S, et al. Spontaneous oligomerization of a staphylococcal alpha-hemolysin conformationally constrained by removal of residues that form the transmembrane beta-barrel. *Protein Eng.* **10**, 1433-1443 (1997).

3. Jo S, Kim T, Iyer VG, & Im W. CHARMM-GUI: a web-based graphical user interface for CHARMM. *J. Comput. Chem.* **29,** 1859-1865 (2008).

4. Gebhardt J, Kleist C, Jakobtorweihen S, & Hansen N. Validation and Comparison of Force Fields for Native Cyclodextrins in Aqueous Solution. *J. Phys. Chem. B* **122,** 1608-1626 (2018).

5. Abraham MJ, *et al.* GROMACS: High performance molecular simulations through multi-level parallelism from laptops to supercomputers. *Software X* **(1–2),** 19–25 (2015).

6. Klauda JB, et al. (2010) Update of the CHARMM all-atom additive force field for lipids: validation on six lipid types. *J. Phys. Chem. B* **114,** 7830-7843 (2010).

7. Darden T, York D, & Pedersen L. Particle mesh Ewald: An N ·log (N) method for Ewald sums in large systems. *J. Chem. Phys.* **98,** 10089–10092 (1993).

8. Hess B, Bekker H, Berendsen HJC & Fraaije JGEM. LINCS: A linear constraint solver for molecular simulations. *J. Comput. Chem.* **18,** 1463–1472 (1997).

9. Evans DJ & Holian BL. The Nose–Hoover thermostat. *J. Chem. Phys.* **83,** 4069–4074 (1985).

10. Aksimentiev A & Schulten K. Imaging alpha-hemolysin with molecular dynamics: ionic conductance, osmotic permeability, and the electrostatic potential map. *Biophys. J* **88,** 3745-3761 (2005).

11. Laskowski RA, MacArthur MW, Moss DS & Thornton JM. PROCHECK: a program to check the stereochemical quality of protein structures, *J. Appl. Crystallogr.* **26**, 283–291(1993).

12. Towse CL, Hopping G, Vulovic I & Daggett V. Nature versus design: the conformational propensities of d-amino acids and the importance of side chain chirality, *Protein Eng. Des. Sel.* **27**, 447–455 (2014).

Source data for SDS gels

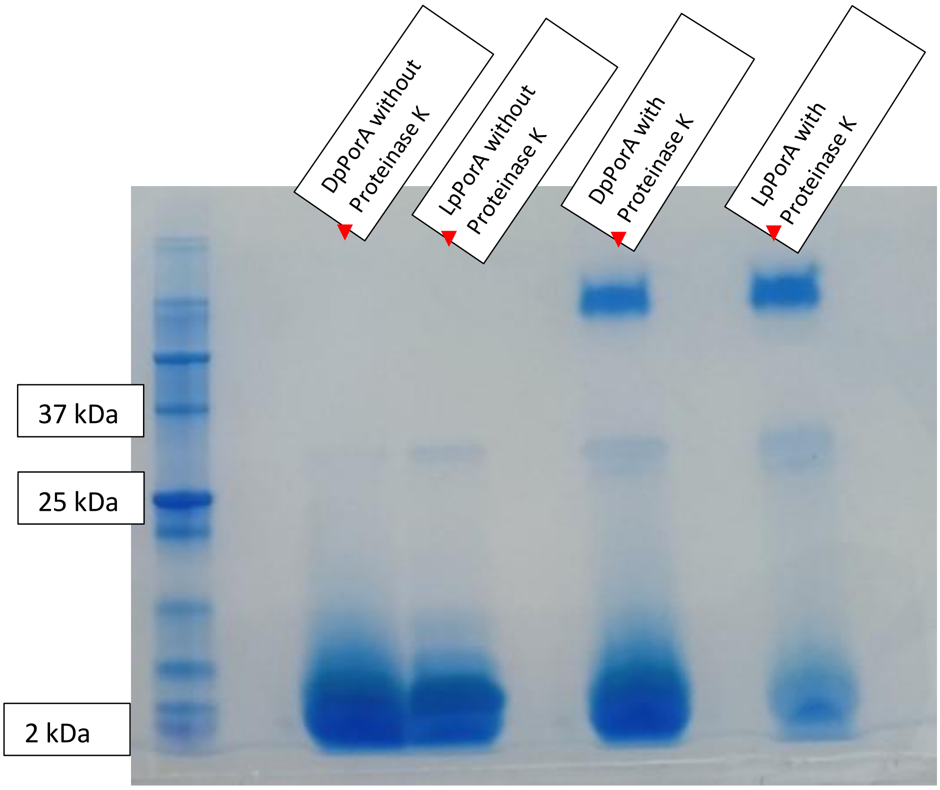

Supplementary Fig. 9a(1)

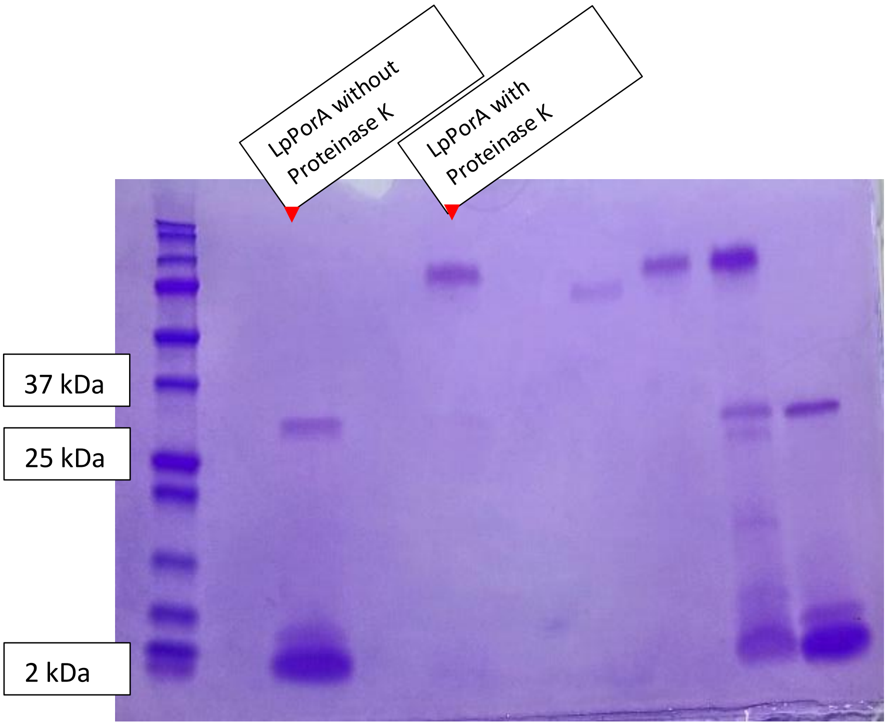

Supplementary Fig. 9a(2)

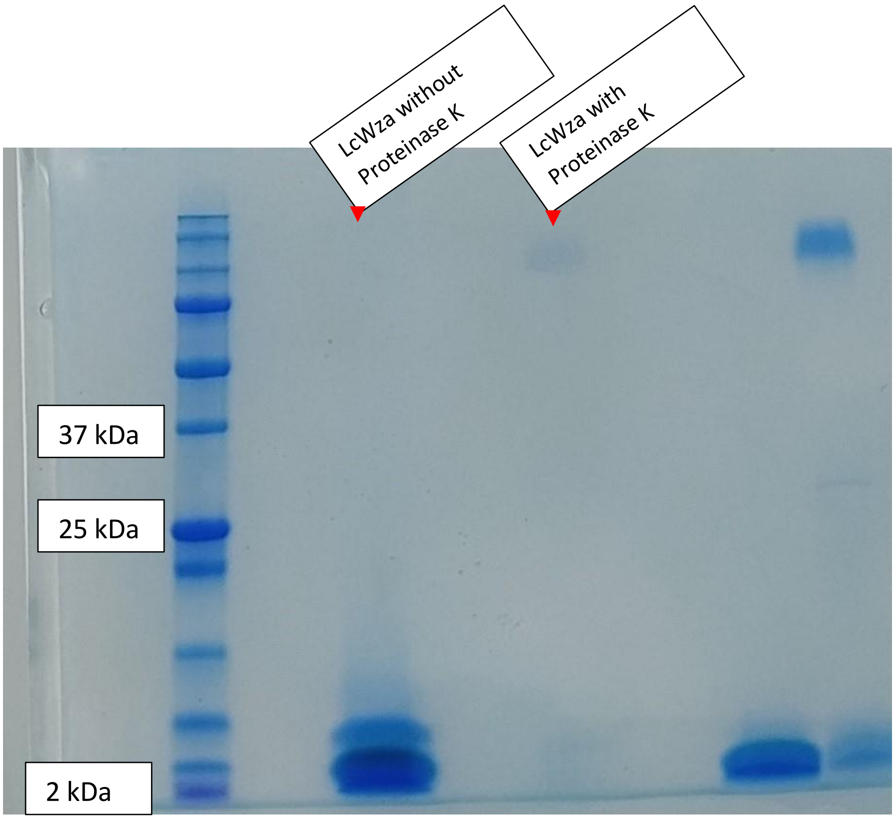

Supplementary Fig. 13b

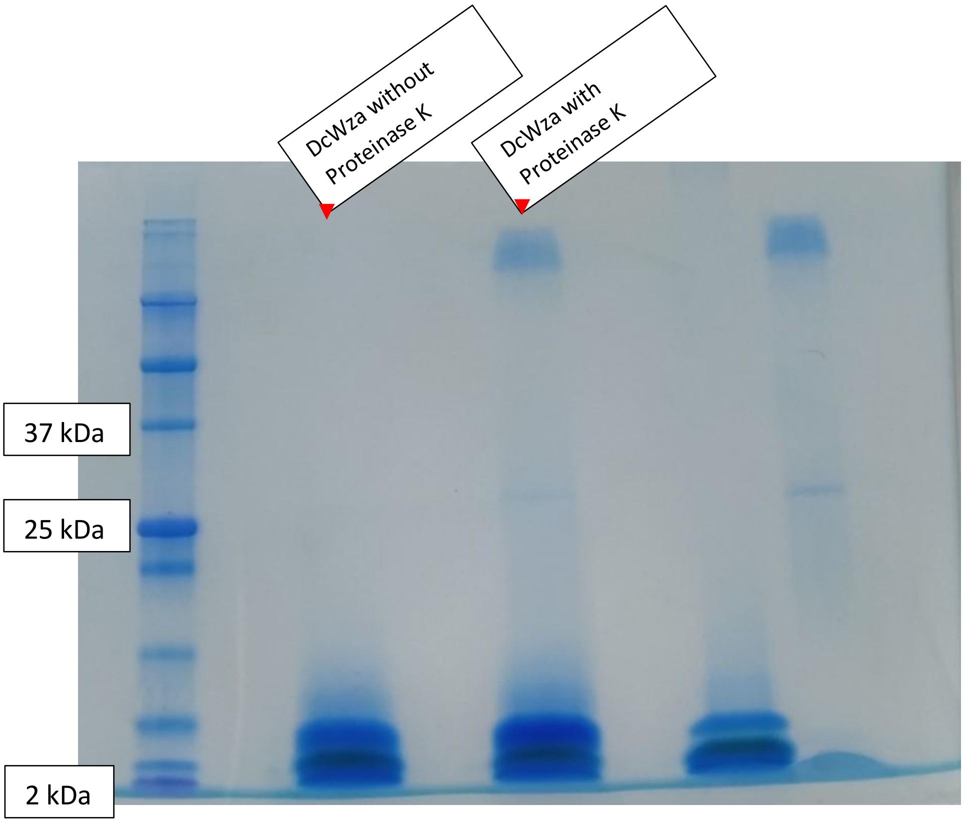

Supplementary Fig. 13c
